# Plastic, synthetic material, or polymer? How material terminology shapes material meanings and informs green technology evaluations

**DOI:** 10.3389/fpsyg.2026.1951034

**Published:** 2026-09-22

**Authors:** Barbara Stiglbauer, Sabrina Schopf

**Affiliations:** 1 Department of Social Psychology, Human Resource Development and Adult Education, Institute of Psychology, Johannes Kepler University Linz, Linz, Austria; 2 Linz Institute for Transformative Change (LIFT_C), Johannes Kepler University Linz, Linz, Austria

**Keywords:** green technology acceptance, material perception, material terminology, plastic, polymer, sustainability

## Abstract

**Introduction:**

Material-related communication plays an important role in sustainability transitions, yet little is known about whether the terms used to describe polymer-based materials shape public evaluations. We investigated whether material terminology influences perceptions of polymer-based materials and whether such terminology-related differences extend to evaluations of green technologies.

**Methods:**

Study 1 (*N* = 303) used a between-subjects design comparing three material terms (plastic, synthetic material, and polymer) that refer to overlapping categories in everyday non-expert communication but are not technically equivalent. Participants evaluated the material on multiple perceptual dimensions and reported their attitude toward its use; exploratory free associations were additionally assessed. Study 2 (*N* = 390) employed a 3 × 2 between-subjects experiment manipulating material terminology and information highlighting the material’s functional and sustainability-related contributions to green technologies. Material and technology perceptions as well as intention to use green technologies were examined, and path analyses examined indirect associations linking the experimental conditions to technology evaluations.

**Results:**

In Study 1, plastic elicited less favourable attitudes toward material use than synthetic material or polymer. These differences were statistically accounted for mainly by ecological and functional-material perceptions. Exploratory free associations converged with the rating-based findings, indicating that plastic more readily evoked ecological harm, whereas polymer elicited more science- and chemistry-related associations as well as greater unfamiliarity. In Study 2, material terminology and information each influenced perceived material sustainability and usefulness, with no evidence that the information attenuated terminology effects. Direct experimental effects on technology perceptions and intention to use were limited, but path analyses showed significant indirect associations through material and technology perceptions, with the strongest indirect associations emerging through perceived usefulness.

**Discussion:**

Together, the findings suggest that material terminology can activate different category representations and multidimensional material meanings and that these meanings may contribute to evaluations of technologies in which the materials are used. Importantly, the sustainability-related outcomes reflect participants’ perceptions rather than objective environmental performance. Communication about polymer-based materials and green technologies should therefore consider both the meanings activated by material terminology and substantiated information about a material’s role and environmental performance in specific applications.

## 1 Introduction

Plastic pollution is a major environmental problem, reflected in increasing production, large volumes of waste and historically low recycling rates (Geyer et al., 2017), leakage into natural environments (Borrelle et al., 2020), and persistent ecological consequences (MacLeod et al., 2021). At the same time, the materials commonly referred to as plastics are polymeric materials whose relevance is not defined by disposability alone. Properties such as low weight, durability, flexibility, barrier performance, insulation, and resistance to environmental stressors make plastic and other polymer-based materials useful across domains such as medicine, transport, construction, packaging, and energy-related technologies (Andrady and Neal, 2009). This tension between environmental harm and functional utility suggests that public evaluations of polymer-based materials are unlikely to reflect technical properties alone. They are also shaped by the meanings people associate with these materials, including concerns about waste and pollution as well as perceptions of usefulness, durability, safety, and technological value.

Prior research likewise indicates that public evaluations of plastics and plastic-based products are multidimensional. People are aware of the environmental problems associated with plastics, but also value their convenience, versatility, affordability, and practicality (Heidbreder et al., 2019). Attitudes toward conventional and bio-based plastics likewise comprise multiple evaluative reactions, including beliefs and emotions related to environmental consequences, usefulness, risk, and the use of plastic and bio-based plastic (Zwicker et al., 2020). Related evidence from bioplastics research further highlights the relevance of knowledge, perceived environmental implications, and end-of-life assumptions for public evaluations (Dilkes-Hoffman et al., 2019). Research on plastic medical devices similarly suggests that preferences for conventional versus bio-based plastic materials are embedded in broader psychological networks of beliefs, emotions, trust, and application-related evaluations (Chambon et al., 2023). Together, this work shows that plastic-related evaluations are multidimensional. However, this research has paid less attention to whether these meanings also depend on the terms used to name the material, especially when everyday language uses different terms for related or partly overlapping material categories.

### 1.1 Material terminology and category representations

Labels and descriptions can function as frames by making certain aspects of an object or issue more salient (Entman, 1993). More broadly, different descriptions can shape interpretation, evaluation, and choice (Tversky and Kahneman, 1981). Related cue-based effects have been documented in environmental and consumer judgement: organic claims can influence health-related judgements (Schuldt and Schwarz, 2010), naturalness cues can shape risk perception and acceptance (Siegrist and Sütterlin, 2017), eco-labels can affect evaluations of otherwise unchanged objects (Sörqvist et al., 2015), and packaging materials and graphics can influence sustainability inferences and product evaluations (Steenis et al., 2017). This broader body of cue-based research has recently been synthesised in a systematic review showing that sustainability cues embedded in product design, including packaging-related cues, can shape user perceptions and product evaluations, although their effects vary across product categories and cue characteristics (Sierra-Fontalvo et al., 2026).

The present studies extend this research by examining the naming of polymer-based material categories. Material terms may do more than denote a category: under limited or ambiguous information, they may make different stored associations, exemplars, and evaluative implications accessible. For example, research on the naming of unfamiliar technologies suggests that names can shape emerging attitudes when people interpret unfamiliar terms through associations with more familiar categories (Boersma et al., 2019). Research on nanotechnology shows that labels can alter perceived risks and benefits of technology-containing products (Siegrist and Keller, 2011), and that risk perceptions are related to trust and vary across nanotechnology applications (Capon et al., 2015).

Throughout this paper, we refer to the associations and evaluative reactions activated by a material term as material meanings. This label does not introduce a new construct. It draws on established multidimensional accounts of material attitudes (e.g., Zwicker et al., 2020) and highlights that such evaluations may depend on which term is used to refer to the material.

We focus on three German material terms that are used in everyday non-expert communication for related and partly overlapping material categories but are not technically equivalent: Plastik, Kunststoff, and Polymer. We use plastic, synthetic material, and polymer as approximate English renderings. Importantly, these terms should not be understood as technical synonyms. In materials science, a polymer is a substance composed of macromolecules (Jenkins et al., 1996) and represents a broader scientific category than plastic, encompassing both naturally occurring polymers, such as cellulose, and synthetic polymers. Plastic, in turn, refers to a polymeric material that may also contain additional substances (International Union of Pure and Applied Chemistry [IUPAC], 2025). Throughout this paper, we use polymer-based materials as an umbrella expression for the partly overlapping referential domain relevant to our lay-language task, not to imply technical equivalence among Plastik, Kunststoff, and Polymer. At the same time, non-experts may not consistently represent these technical category boundaries. We therefore treat the three terms as cues to partly overlapping category representations that differ in semantic register—colloquial, formal, and technical—and may consequently make different associations, exemplars, and evaluative implications accessible. Thus, the present study is not a pure relabelling experiment in which an objectively identical material was presented under three technically interchangeable names. Rather, it examines the effects of material terminology together with the category representations evoked by those terms when the specific physicochemical material remains unspecified.

We expected Plastik to function as the most colloquial of the three terms and therefore to evoke comparatively accessible everyday associations with consumer products, packaging, disposability, waste, and pollution. We expected Kunststoff to evoke a somewhat more formal representation; literally translated, the term denotes a human-made or synthetic material. Polymer was expected to be less familiar and evoke the most abstract and technical material-science representation. These terminological distinctions may be psychologically consequential, yet existing research leaves a specific gap. Research on plastics has primarily examined material perceptions, attitudes, preferences, and consumption-reduction interventions, whereas research on environmental labelling has concentrated on explicit claims, certifications, naturalness cues, or packaging information. Furthermore, sustainable-energy acceptance research has identified perceived benefits, costs, risks, affect, trust, fairness, and perceived behavioural control as relevant determinants of technology attitudes and intentions (Huijts et al., 2012; Perlaviciute and Steg, 2014), while critical reviews emphasise the importance of broader social and contextual factors (Batel, 2018, 2020). What remains less clear is whether basic material terminology itself functions as an antecedent of multidimensional material meanings and whether these meanings remain confined to the material level or extend to evaluations of technologies in which the material is embedded.

### 1.2 The present research

Against this background, the present research examined whether material terminology shapes laypeople’s sustainability-related perceptions and evaluations by activating different material meanings, and whether these meanings are associated with evaluations of green technologies in which such materials are used. Study 1 tested whether the three terms affect attitudes toward material use and whether observed differences are statistically accounted for by term-activated material meanings. Study 2 examined material terminology and information highlighting functional and sustainability-related benefits of the material for its application in green technologies as distinct cues that shape material perceptions and may be associated with technology evaluations through indirect pathways. Importantly, sustainability-related outcomes throughout the paper refer to participants’ subjective perceptions and evaluations and should not be interpreted as evidence of objective material properties or environmental performance.

## 2 Study 1

Study 1 examined whether describing related or partly overlapping material categories as plastic, synthetic material, or polymer affects attitudes toward material use and the meanings people attach to the material. We assumed that such terminology effects would not be limited to global evaluation. Prior research suggests that public evaluations of plastics are ambivalent and multidimensional: people are aware of plastic-related environmental problems, but also value plastics for their convenience, practicality, and usefulness (Heidbreder et al., 2019). Attitudes toward conventional and bio-based plastics likewise comprise multiple evaluative reactions, including beliefs and emotions related to environmental consequences, usefulness, risk, and use (Zwicker et al., 2020). We therefore focused on three evaluative material-meaning dimensions that capture central ecological, functional, and risk- or safety-related meanings. Because the terms also differ in semantic register and expected lay familiarity, we included familiarity as an additional focal dimension.

Ecological value captured whether the material was perceived as environmentally responsible, environmentally friendly, and sustainable. This dimension was intended to assess a broad ecological evaluation of the labelled material, motivated by prior work documenting plastic-related environmental problems such as waste, pollution, leakage into natural environments, and persistent ecological consequences (Borrelle et al., 2020; Geyer et al., 2017; MacLeod et al., 2021), as well as public awareness of these problems (Heidbreder et al., 2019).

Functional-material value captured whether the material was perceived as useful, valuable, and innovative. This dimension reflects the functional side of plastic-related ambivalence: although plastics are associated with environmental problems, they are also valued for convenience, practicality, and usefulness in everyday life (Heidbreder et al., 2019; Zwicker et al., 2020). At the material level, polymer-based materials provide properties such as versatility, durability, low weight, and technological performance, which make them useful across a wide range of applications (Andrady and Neal, 2009). Because this dimension combined usefulness, value, and innovativeness, it was interpreted as a broad functional-material evaluation rather than as perceived usefulness alone.

We further included safety-related material impression because material terms may evoke broader impressions of cleanliness, danger, and trustworthiness. For example, risk-perception research shows that evaluations of uncertain or contested hazards are shaped by perceived risk, affect, and trust in relevant actors (Siegrist and Cvetkovich, 2000; Slovic, 1987). Furthermore, research on products made from recycled plastic bottles has demonstrated a contact-related contamination response (Meng and Leary, 2021): Such products were perceived as contaminated, particularly when they involved skin contact (e.g., T-shirt as opposed to a bag), which in turn reduced consumers’ purchase intentions. In our study, safety-related material impression therefore captured whether the material appeared clean, safe, and trustworthy. This composite should be understood as a broad evaluative impression rather than as a specific measure of personal, technical, or environmental safety.

Finally, we included familiarity because the terms differ in semantic register and expected lay recognisability. We expected plastic, as a colloquial everyday term, to be more familiar to laypeople than polymer, which has a more technical register and denotes a broader scientific category (Jenkins et al., 1996). Familiarity was therefore included as a dimension conceptually distinct from ecological, functional, and safety-related evaluations.

Because material terms may evoke broad meaning profiles, participants evaluated the material on a semantic differential covering 15 adjective pairs. This broader item pool allowed us to characterise the overall profiles elicited by the three terms. For the focal hypothesis tests, however, we concentrated on four dimensions derived from the preceding theoretical rationale: ecological value, functional-material value, safety-related material impression, and familiarity. Additional facets were retained to describe broader term-evoked profiles and to assess the robustness of the main findings, but were not treated as primary hypothesis-relevant dimensions.

We first tested whether attitudes toward using the material differed depending on whether the material was described as plastic, synthetic material, or polymer. First of all, we expected plastic to elicit less favourable attitudes toward material use than synthetic material and polymer (H1). Prior research indicates that plastic is especially salient in everyday consumer contexts such as packaging and single-use products, and public perceptions of plastic are strongly structured by concerns about waste, pollution, persistence, and environmental harm (Heidbreder et al., 2019; Henderson, 2023; Zwicker et al., 2020). As plastic is the most colloquial of the three terms, it is likely that this term activates these, predominantly, negative associations more strongly than the more formal or technical terms.

We then examined whether the terms differed in material-perception dimensions (H2). We expected plastic to elicit lower ecological value than synthetic material and polymer because it was expected to activate pollution-, waste-, and environmental-harm associations more readily (H2a). We also expected polymer to elicit lower familiarity than plastic and synthetic material because it is more technical and less common in everyday language (H2b). For functional-material value and safety-related material impression, we expected term-based differences because the terms may activate different functional, technical, contamination-related, and trust-related associations. However, the existing literature did not support sufficiently clear directional predictions. We therefore did not specify the direction of the expected differences in functional-material value (H2c) or safety-related material impression (H2d).

If differences in attitudes toward material use are linked to the meanings activated by the terms, these differences should be statistically accounted for by material perceptions (H3). We expected ecological value and functional-material value to be particularly relevant because they represent the central environmental and functional meanings of polymer-based materials. Safety-related material impression and familiarity were included as additional material-perception dimensions that may further contribute to explaining term-based attitude differences.

### 2.1 Materials and methods

#### 2.1.1 Study design and procedure

Study 1 used a one-factor between-subjects experimental design with three material-term conditions: Plastik (“plastic”), Kunststoff (“synthetic material”), and Polymer (“polymer”). Participants were recruited through the professional German online panel Bilendi and completed an anonymous online survey. They received panel-provider bonus points according to the provider’s standard procedures. The questionnaire was administered in German and identical across conditions except that all material-related questions referred to the assigned term. No specific chemical composition, polymer type, product, or application was specified. Thus, the experimental manipulation varied the material term while leaving the concrete material referent open. After demographic questions, participants completed an exploratory free-association task. They then evaluated the assigned material term on a semantic differential and completed the attitude toward material use scale and additional individual-difference measures.

All procedures were conducted in accordance with applicable laws and institutional guidelines and with the ethical principles of the Declaration of Helsinki: Participation was voluntary, and before accessing the questionnaire, participants received information about the study purpose and procedures, anonymous data handling, their right to discontinue participation without disadvantage, and contact details for the research team. All participants provided electronic informed consent before beginning the survey and were debriefed upon completion. The research team received no directly identifying participant information, and participants’ privacy rights were protected throughout the research.

Target sample sizes were determined *a priori* using G*Power Version 3.1.9.7 (Faul et al., 2009) to provide 80% power at α = 0.05 to detect small-to-medium experimental effects of Cohen’s f = 0.18. In addition to the panel provider’s quality-control procedures, study-specific data-quality screening was applied before analysis following recommendations for identifying careless and low-quality responses in online survey data (Curran, 2016; DeSimone et al., 2015; Meade and Craig, 2012; Zhang and Conrad, 2014). Detailed power-analysis parameters, screening rules, and details regarding the semantic differential measure are provided in the Supplementary Sections A–C.

#### 2.1.2 Participants

A total of *N* = 330 participants completed the survey. Based on the screening criteria, 27 participants were excluded, resulting in a final analytic sample of 303 participants. The final sample included 101 participants in the plastic condition, 104 in the synthetic material condition, and 98 in the polymer condition. Participants were between 18 and 69 years old (*M* = 39.43, *SD* = 14.95); 57.1% identified as men and 42.9% as women. In total, 42.2% reported having children. Regarding education, 6.3% reported compulsory schooling as their highest educational attainment, 39.9% vocational training without university entrance qualification, 19.8% university entrance qualification, 31.0% a university degree, and 3.0% another or no formal qualification.

#### 2.1.3 Measures

First, participants completed an exploratory free-association task intended to capture spontaneously accessible material meanings in their own words. The task was developed in line with prior work on public perceptions of plastics and material preferences (Chambon et al., 2023). Participants were presented with their assigned material term and asked to spontaneously report thoughts, feelings, impressions, individual words, or short phrases that came to mind. The instructions emphasised that there were no right or wrong answers and that participants should report their first spontaneous associations without deliberating extensively. Participants could provide up to seven associations and were encouraged to provide at least one or two.

Material perceptions were assessed using a semantic differential format (Osgood et al., 1957). The item pool was informed by prior research on multidimensional plastic-related attitudes (Heidbreder et al., 2019; Zwicker et al., 2020) and refined in collaboration with experts in polymer and materials science. Participants evaluated the assigned material term on 15 bipolar adjective pairs using 5-point scales. Importantly, responses represent laypeople’s subjective perceptions and not objective material properties. For the focal analyses, semantically related adjective pairs were averaged into the theory-driven composite indices: ecological value (not sustainable–sustainable, environmentally harmful–environmentally friendly, irresponsible–responsible; α = 0.87), functional-material value (useless–useful, low value–high value, outdated–innovative; α = 0.69), and safety-related material impression (dangerous–safe, not trustworthy–trustworthy, dirty–clean; α = 0.77). Familiarity was assessed with the single item unfamiliar–familiar. Additional semantic-differential facets were retained to characterise broader term-evoked meaning profiles and to assess robustness: Overall evaluation was calculated as the mean of the two broad evaluative items bad–good and unattractive–attractive (α = 0.73). Naturalness (artificial–natural), longevity (short-lived–long-lasting), and complexity (complex–simple) were analysed as additional single-item facets. The complete item rationale and robustness analyses are reported in the Supplementary Sections C, E.

Attitude toward material use was assessed with four bipolar items adapted from Venkatesh et al. (2003). Participants indicated whether they considered the use of the assigned material to be a bad idea versus good idea, unwise versus wise, something they disliked versus liked, and something that gave them a bad feeling versus good feeling. Items were assessed on 7-point scales, with higher values indicating a more favourable attitude toward material use. Internal consistency was high (α = 0.94).

#### 2.1.4 Statistical analyses

Statistical analyses were conducted using IBM SPSS Statistics for Windows, Version 29.0 (IBM Corp, 2022b). To test whether material terms affected attitudes toward material use (H1), a one-way analysis of variance with term condition as the between-subjects factor and attitude toward material use as the dependent variable was conducted. Differences in material perceptions (H2) were examined using a multivariate analysis of variance with term condition as the between-subjects factor and ecological value, functional-material value, safety-related material impression, and familiarity as dependent variables. Significant omnibus effects were followed by univariate tests and Games–Howell *post hoc* comparisons.

To examine whether material perceptions statistically accounted for terminology differences in attitude toward material use (H3), we estimated a parallel mediation model using PROCESS Model 4 (Hayes, 2022), with attitude toward material use as the outcome. Term condition was entered as a categorical predictor with plastic as the reference category, yielding two relative contrasts: synthetic material versus plastic and polymer versus plastic. Indirect effects were estimated using 10,000 bootstrap samples and 95% bias-corrected bootstrap confidence intervals.

The free-association analysis was exploratory. Each non-empty association field was treated as a separate coding unit and assigned to one of 29 mutually exclusive codes. Two coders independently coded all 863 associations using the final coding framework. Exact agreement between the two independent coders was 94.0% (Cohen’s κ = 0.936). Disagreements were subsequently resolved by consensus. For analysis, the consensus codes were aggregated to binary participant-level indicators reflecting whether each category was mentioned at least once. The number of associations provided was compared across conditions using a Kruskal–Wallis test with Holm-adjusted pairwise Mann–Whitney U-tests. Category frequencies were compared using Pearson chi-square tests or two-sided Fisher–Freeman–Halton exact tests for sparse tables. Omnibus *p*-values across the 28 comparisons were adjusted using the Benjamini–Hochberg false-discovery-rate procedure (Benjamini and Hochberg, 1995); significant adjusted omnibus effects were followed by two-sided Fisher exact tests with Holm adjustment within category. A sensitivity analysis repeated the category comparisons using only each participant’s first association. Full details are reported in the Supplementary Section F.

### 2.2 Results

Descriptive statistics for the focal outcomes by material-term condition, along with the corresponding univariate analyses of variance, are presented in Table 1. Item-level semantic-differential profiles and full pairwise comparisons are reported in the Supplementary Sections C, D, respectively.

**TABLE 1:** Descriptive statistics and terminology effects for attitude toward material use and material perceptions in study 1.

	Descriptive statistics: *M* (*SD*)			Terminology effects: ANOVA			
Variable	Plastic	Synthetic material	Polymer	*F*	*df*	*p*	Partial η^2^
Attitude toward use	3.46^a^ (1.46)	4.12^b^ (1.28)	3.97^b^ (1.25)	6.89	2, 300	0.001	0.044
Ecological value	2.06^a^ (0.99)	2.51^b^ (1.02)	2.73^b^ (0.90)	12.10	2, 300	< 0.001	0.075
Functional-material value	3.07^a^ (0.82)	3.39^b^ (0.79)	3.42^b^ (0.82)	5.77	2, 300	0.004	0.037
Safety-related material impression	2.75^a^ (0.98)	3.24^b^ (0.85)	3.21^b^ (0.90)	9.25	2, 300	< 0.001	0.058
Familiarity	4.05^a^ (0.99)	4.10^a^ (0.94)	2.86^b^ (1.17)	45.75	2, 300	< 0.001	0.234

Means with different superscripts differ significantly at *p* < 0.05 based on Games-Howell *post hoc* tests. Semantic-differential dimensions were assessed on 5-point scales; attitude toward use was assessed on a 7-point scale.

#### 2.2.1 Terminology effects on attitude toward material use and material perceptions

The one-way ANOVA testing H1 showed a significant effect of material term on attitude toward material use, *F*(2, 300) = 6.89, *p* = 0.001, partial η^2^ = 0.044. Games–Howell comparisons showed that attitudes toward material use were less favourable for plastic than for synthetic material, *M*_*diff*_ = −0.66, *SE* = 0.19, 95% CI [−1.11, −0.21], *p* = 0.002, and polymer, *M*_*diff*_ = −0.51, *SE* = 0.19, 95% CI [−0.96, −0.05], *p* = 0.024. Synthetic material and polymer did not differ significantly, *M*_*diff*_ = 0.15, *SE* = 0.18, 95% CI [−0.27, 0.57], *p* = 0.665. Thus, H1 was supported.

The significant multivariate effect of material term, Pillai’s trace = 0.355, *F*(8, 596) = 16.06, *p* < 0.001, partial η^2^ = 0.177, indicated distinct material-perception profiles. Univariate analyses showed significant effects for all four focal dimensions (Table 1). The predicted differences in ecological value (H2a) and familiarity (H2b) were supported: ecological value was lower in the plastic condition than in the synthetic material and polymer conditions, whereas polymer was substantially less familiar than both other terms. Significant differences also emerged for functional-material value (H2c) and safety-related material impression (H2d), with less favourable ratings in the plastic condition than in the other two conditions.

#### 2.2.2 Indirect effects through material perceptions

The parallel mediation model explained substantial variance in attitude toward material use, *R*^2^ = 0.556, *F*(6, 296) = 61.76, *p* < 0.001. Ecological value was positively associated with attitude toward material use, *B* = 0.44, *SE* = 0.07, 95% CI [0.29, 0.58], *p* < 0.001. Functional-material value was also positively associated with attitude toward use, *B* = 0.51, *SE* = 0.09, 95% CI [0.34, 0.69], *p* < 0.001, as was safety-related material impression, *B* = 0.34, *SE* = 0.09, 95% CI [0.16, 0.51], *p* < 0.001. Familiarity, in contrast, did not significantly relate to attitude toward material use, *B* = −0.003, *SE* = 0.06, 95% CI [-0.11, 0.11], *p* = 0.959. After including the material-perception mediators, the direct effects of terminology were no longer statistically significant, Δ*R*^2^ = 0.005, *F*(2, 296) = 1.59, *p* = 0.206.

Bootstrapped indirect effects are reported in Table 2. For both material term contrasts, synthetic material versus plastic and polymer versus plastic, significant indirect effects emerged through ecological value, functional-material value, and safety-related material impression. Indirect effects through familiarity were not significant. The pattern was therefore consistent with H3: term differences in attitudes toward material use were statistically accounted for by multiple material perceptions. Ecological value and functional-material value provided the most central pathways.

**TABLE 2:** Relative indirect effects of material term on attitude toward material use (study 1).

Contrast	Mediator	Indirect effect	*SE*	95% CI
Synthetic material vs. plastic	Ecological value	0.20	0.07	[0.07, 0.37]
	Functional-material value	0.17	0.07	[0.05, 0.33]
	Safety-related material impression	0.17	0.06	[0.07, 0.32]
	Familiarity	−0.00	0.01	[−0.02, 0.02]
Polymer vs. plastic	Ecological value	0.29	0.08	[0.15, 0.48]
	Functional-material value	0.18	0.07	[0.06, 0.35]
	Safety-related material impression	0.15	0.06	[0.06, 0.30]
	Familiarity	0.00	0.08	[−0.15, 0.15]

Values are unstandardised indirect effects with 95% bias-corrected bootstrap confidence intervals. Confidence intervals were based on 10,000 bootstrap samples.

#### 2.2.3 Robustness and exploratory analyses

Robustness analyses are reported in the Supplementary Section E. First, the mediation model was repeated including overall evaluation as an additional mediator. Because overall evaluation captured a broad affective-evaluative impression conceptually close to attitude toward material use, it was not included in the focal model. Including it did not alter the substantive conclusion that terminology effects on attitudes toward material use were accounted for by material perceptions, although overall evaluation absorbed part of the shared variance. Second, additional semantic-differential facets that were not part of the composite dimensions, including naturalness, longevity, and complexity, were examined in expanded robustness models. The conclusions remained unchanged, indicating that our findings were not driven by these additional material-related facets.

Finally, the exploratory free-association analysis provided convergent evidence regarding spontaneously accessible material meanings. Negative environmental meanings were most frequent for plastic (56.4%), followed by synthetic material (27.9%) and polymer (7.1%), χ^2^(2, *N* = 303) = 57.40, *p* < 0.001, Cramér’s *V* = 0.435; all three conditions differed in Holm-adjusted pairwise comparisons. Science- and chemistry-related terminology showed the reverse pattern, occurring for 1.0% of participants in the plastic condition, 2.9% in the synthetic-material condition, and 18.4% in the polymer condition, χ^2^(2, *N* = 303) = 26.81, *p* < 0.001, Cramér’s *V* = 0.297. Unfamiliarity or lack of an accessible association was likewise concentrated in the polymer condition (20.4%) relative to plastic (1.0%) and synthetic material (1.9%), χ^2^(2, *N* = 303) = 33.99, *p* < 0.001, Cramér’s *V* = 0.335. Because participants in the polymer condition generated fewer associations overall, the analyses were repeated using only each participant’s first association. The reported contrasts remained significant after Benjamini–Hochberg adjustment. Complete category frequencies, adjusted omnibus tests, pairwise comparisons, and sensitivity analyses are reported in the Supplementary Section F.

## 3 Study 2

Study 2 examined whether the meanings activated by different material terms extend beyond material evaluations and inform evaluations of green technologies. Many technologies involved in sustainability transitions rely on specific material properties, yet lay evaluations of these technologies are unlikely to be based on detailed knowledge of their components, production processes, or life-cycle implications. Materials may therefore serve as additional interpretive cues. If a material is perceived as environmentally problematic or low in functional value, these meanings may colour evaluations of technologies in which it is used. Conversely, if the material is perceived as functional and compatible with sustainability goals, it may support more favourable technology evaluations.

We conceptualise this possibility as an inferential material-to-technology transfer of meaning. When people have limited knowledge of how a material functions within a technology, they may use material perceptions as inferential cues when judging the broader technology. Prior research shows that environmental labels and packaging-related cues can influence evaluations beyond the attribute directly indicated by the cue (Sörqvist et al., 2015; Steenis et al., 2017). Applied to green technologies, the perceived meaning of a material may therefore become part of the interpretive context for evaluating the technology in which it is used.

Two transfer pathways were especially relevant because they linked the two material perceptions most central to a green-technology context with corresponding technology-level evaluations. The sustainability pathway linked perceived material sustainability (PMS) with perceived technology sustainability (PTS): sustainability-related meanings attached to the material may inform whether technologies using it are seen as environmentally beneficial or compatible with sustainability goals. The usefulness pathway linked perceived material usefulness (PMU) with perceived technology usefulness (PTU): perceived material functionality can signal performance, durability, reliability, and contribution to the technology’s functioning. At the technology level, PTU was expected to be particularly important for intention to use (ITU), given the central role of perceived usefulness in technology-acceptance models (Davis, 1989; Venkatesh et al., 2003). PTS was also expected to be relevant because environmental benefit is central to the value proposition of green technologies. These expectations are consistent with sustainable-energy technology acceptance frameworks (Huijts et al., 2012; Perlaviciute and Steg, 2014) and evidence that perceived benefits, including environmental advantages, are relevant to low-carbon technology attitudes and adoption intentions (Scheller et al., 2024).

Material terminology is one source of material meaning, but it is not the only one. Evaluations may also depend on information that links material properties to application-specific environmental and functional contributions. Life-cycle and circular-economy research emphasises that environmental implications of polymer-based materials depend on life-cycle stage, functional role, lifetime, and end-of-life assumptions rather than on material category alone (Jiao et al., 2024; Nordahl and Scown, 2024; Pellengahr et al., 2023). A material associated with environmental problems in disposable packaging may therefore have different implications when it contributes to durability, efficiency, or weight reduction in long-lived technologies. In the present study, we therefore compared two information conditions: Green-technology-specific benefit information, which highlighted favourable functional properties and linked these properties to the functioning and sustainability-related benefits of green technologies, and general application information, which described material forms and technical uses without making the same functional and sustainability-related links and served as control condition.

Against this background, Study 2 examined whether material terminology and information condition shape PMS and PMU, and whether these perceptions are associated with technology-level evaluations (PTS and PTU) and ITU. Participants received one of the three material terms and either the green-technology-specific benefit information or the general application information. Based on the terminology argument and Study 1, we expected material term to affect PMS and PMU (H4). More specifically, polymer was expected to elicit higher PMS and PMU than plastic, because its more technical connotation was expected to make waste- and pollution-related associations less immediately accessible and to fit technical applications more readily. Synthetic material was expected to occupy an intermediate conceptual position, but its exact position relative to plastic and polymer was examined empirically.

We further expected green-technology-specific benefit information to increase PMS and PMU (H5): The text in this condition made favourable functional contributions and sustainability-related implications salient rather than merely providing additional neutral context. Thus, this type of information should shift attention from generic material associations to functional and sustainability-relevant aspects of the material in green technologies. Whether this information would attenuate term-based differences was examined as an open question (RQ1). The information might reduce reliance on terminology, but everyday associations, especially those linked to plastic, may also persist.

Finally, Study 2 tested the proposed material-to-technology transfer process. We expected PMS to be positively associated with PTS, and PMU to be positively associated with PTU. PTS and PTU were, in turn, expected to be positively associated with ITU (H6). Accordingly, we expected material term and information condition to be indirectly associated with ITU through material perceptions and technology perceptions (H7). Because the experimental manipulations directly targeted the material rather than the technology itself, total effects on technology perceptions and ITU were expected to be weaker than effects on material perceptions. We therefore examined total experimental effects on these more distal outcomes exploratorily (RQ2).

### 3.1 Materials and methods

#### 3.1.1 Study design and procedure

Study 2 used a 3 × 2 between-person experimental survey design. Participants were randomly assigned to one of three material-term conditions [Plastik (“plastic”), Kunststoff (“synthetic material”), or Polymer (“polymer”)] and one of two information conditions (green-technology-specific benefit information vs. general application information). In the green-technology-specific benefit information condition, participants read a short text explaining that the respective material is used in green technologies such as solar panels and wind turbines because of properties such as durability, stability, weather resistance, low weight, and technological versatility. The text explicitly linked these properties to safe and efficient long-term functioning and to sustainable resource use. Thus, this condition provided positively valenced functional and sustainability-related information rather than merely additional neutral context. The general application information served as comparison text. It was similar in length and covered the material’s general application in neutral form. Thus, it described the material’s forms and general technical and industrial applications, including its use in green technologies, but did not explain how specific material properties contribute to the functioning or sustainability of those technologies. The informational content within each information condition was held constant across material-term conditions; no specific chemical composition or polymer type was identified. The texts were developed with input from polymer experts to support scientific accuracy and were reviewed for comprehensibility. Full texts are provided in the Supplementary Section G.

As in Study 1, participants were recruited through the professional German online panel Bilendi. Participants first provided sociodemographic information and were then randomly assigned to one of the six experimental conditions. After reading the manipulation text, they completed an attention check, a text-comprehension check, and an open material manipulation check. They then answered questions about the material, green technologies using the material, and intention to use green technologies. Participants who failed the forced-choice comprehension check were not allowed to continue the survey.

The ethics and informed-consent procedures were identical to those described for Study 1. Sample size planning and data screening also followed the same general logic as in Study 1 (see Supplementary Sections A, B).

#### 3.1.2 Participants

A total of 440 participants completed the survey. Based on the screening criteria, 50 participants were excluded, resulting in a final analytic sample of 390 participants. The final sample included 183 men (46.9%) and 207 women (53.1%). Participants ranged in age from 18 to 69 years (*M* = 42.57, *SD* = 14.60), and 172 participants (44.1%) reported having children. Regarding education, 3.3% reported compulsory schooling as their highest educational attainment, 41.0% vocational training, 21.3% university entrance qualification, 14.6% a bachelor’s degree, 17.7% a master’s or diploma degree, 0.8% a doctoral degree, and 1.3% another degree.

At the end of the survey, participants indicated whether they had already used or installed one or more green technologies. Overall, 129 participants (33.1%) reported prior use or installation of at least one green technology. The most frequently reported technology was photovoltaic systems (*n* = 84, 21.5%), followed by heat pumps (*n* = 46, 11.8%), solar thermal systems (*n* = 42, 10.8%), other technologies (*n* = 11, 2.8%), and wind power participation (*n* = 4, 1.0%). Robustness analyses controlling for prior green-technology use are reported in the Supplementary Sections I, J.

#### 3.1.3 Measures

Sustainability perceptions (PMS and PTS) were assessed with four parallel items adapted from Hamari et al.’s (2016) perceived sustainability scale. Items captured perceived sustainability, compatibility with long-term ecological goals, environmental friendliness, and conservation of natural resources. Material-related items referred to the assigned material term, whereas technology-related items referred to green technologies using the material. Items were rated on the same 7-point scale. Internal consistencies were high for perceived material sustainability (α = 0.92) and perceived technology sustainability (α = 0.94). Importantly, these measures capture participants’ perceived sustainability of the material and technology; they do not assess actual environmental performance or life-cycle sustainability.

Usefulness perceptions (PMU and PTU) were assessed with four parallel items adapted to the present material and technology context from technology acceptance research (Venkatesh et al., 2003). PMU items assessed whether the assigned material was perceived as useful in green technologies, improved the performance of such technologies, supported efficient functioning, and enabled effective use. PTU items assessed whether green technologies were perceived as useful, improved energy generation, contributed to efficient energy generation, and enabled an effective supply of energy. Items were rated on 7-point scales ranging from 1 (strongly disagree) to 7 (strongly agree). Internal consistency was high for both, PMU (α = 0.93) and PTU (α = 0.95).

Intention to use (ITU) was assessed with three items adapted from the Intention to Use subscale of the Technology Usage Inventory (Kothgassner et al., 2012). Participants indicated whether they would like to use green technologies, whether they would want to have access to green technologies, and whether they would support the acquisition of green technologies. Items were rated on a 10-point scale ranging from 1 (does not apply) to 10 (applies). Internal consistency was high (α = 0.94).

#### 3.1.4 Statistical analyses

Statistical analyses were conducted using IBM SPSS Statistics and IBM SPSS Amos for Windows, Version 29.0 (IBM Corp, 2022a,b). As a preliminary assessment of the measurement model, we examined the factorial distinctiveness of the five measured constructs. The five-factor model showed acceptable fit, CFI = 0.960, TLI = 0.952, RMSEA = 0.076, SRMR = 0.041, and fitted the data better than more restrictive models in which conceptually related constructs were combined. Full measurement-model results are reported in the Supplementary Section H.

To test direct experimental effects on proximal material perceptions, a 3 × 2 MANOVA was conducted with material term (H4), information condition (H5), and their interaction (RQ1) as predictors, and PMS and PMU as dependent variables. Significant multivariate effects were followed by univariate ANOVAs and Sidak-corrected pairwise comparisons. To explore whether direct experimental effects were also visible at more distal levels (RQ2), we conducted a second 3 × 2 MANOVA with PTS and PTU as dependent variables, followed by a 3 × 2 ANOVA for ITU.

To test the proposed material-to-technology transfer process (H6–H7), we estimated path models using maximum likelihood estimation. Material term was represented by two dummy variables, with plastic as the reference category. Information condition was coded so that the green-technology-specific benefit information condition was compared with the general application information condition. The hypothesised model specified indirect pathways from the experimental predictors to ITU through material and technology perceptions; direct paths from material perceptions to ITU were retained to account for residual associations beyond technology perceptions. Interaction terms were not included because the factorial analyses showed no evidence that information condition moderated terminology effects on the proximal material perceptions. The path model was therefore designed to test the main-effect transfer pathways implied by H6 and H7. Indirect effects were evaluated using 5,000 bootstrap samples and 95% bias-corrected bootstrap confidence intervals. Because the hypothesised model required a data-informed extension, we distinguish below between the hypothesised, extended, and reduced final models. Full specifications and comparisons are reported in the Supplementary Section J.

### 3.2 Results

Table 3 reports descriptive statistics for the measured variables across the six experimental conditions. Across both information conditions, PMS was lowest in the plastic conditions, higher in the synthetic material conditions, and highest in the polymer conditions. A similar, though less pronounced, pattern emerged for PMU. Descriptively, the green-technology-specific benefit information conditions tended to show slightly higher PMS and PMU than the corresponding general application information conditions across all three material terms. Differences across conditions were smaller for PTS, PTU, and ITU.

**TABLE 3:** Means (standard deviations) across the six experimental conditions in study 2.

Variable	Plastic		Synthetic material		Polymer	
	Green-technology-specific benefit information *n* = 62	General application information *n* = 63	Green-technology-specific benefit information *n* = 72	General application information *n* = 71	Green-technology-specific benefit information *n* = 70	General application information *n* = 52
Material perceptions:						
Sustainability	3.34 (1.49)	3.05 (1.29)	3.92 (1.53)	3.26 (1.45)	4.84 (1.27)	4.56 (1.41)
Usefulness	4.69 (1.36)	4.34 (1.30)	5.08 (1.30)	4.66 (1.37)	5.39 (1.15)	5.17 (1.00)
Technology perceptions:						
Sustainability	5.27 (1.31)	4.75 (1.60)	5.36 (1.43)	5.06 (1.56)	5.33 (1.32)	5.27 (1.44)
Usefulness	5.60 (1.31)	5.00 (1.54)	5.66 (1.34)	5.49 (1.27)	5.48 (1.40)	5.40 (1.26)
Intention to use	7.88 (2.19)	7.19 (2.22)	7.95 (1.91)	7.34 (2.22)	7.50 (2.42)	8.04 (2.07)

Material and technology perception variables were measured on 7-point scales; intention to use was measured on a 10-point scale.

#### 3.2.1 Direct experimental effects

The 3 × 2 MANOVA with PMS and PMU as dependent variables showed significant multivariate effects of material term, Pillai’s trace = 0.17, *F*(4, 768) = 17.53, *p* < 0.001, partial η^2^ = 0.084, and information condition, Pillai’s trace = 0.02, *F*(2, 383) = 4.72, *p* = 0.009, partial η^2^ = 0.024. The term × information interaction was not significant, Pillai’s trace = 0.01, *F*(4, 768) = 0.50, *p* = 0.739, partial η^2^ = 0.003. No significant multivariate direct experimental effects emerged at the technology level. The 3 × 2 MANOVA with PTS and PTU as dependent variables showed no significant multivariate effect of material term, Pillai’s trace = 0.018, *F*(4, 768) = 1.70, *p* = 0.147, partial η^2^ = 0.009, information condition, Pillai’s trace = 0.012, *F*(2, 383) = 2.26, *p* = 0.106, partial η^2^ = 0.012, or their interaction, Pillai’s trace = 0.009, *F*(4, 768) = 0.88, *p* = 0.476, partial η^2^ = 0.005.

Follow-up univariate tests are reported in Table 4. There were significant effects of material term on both PMS and PMU. Polymer was perceived as more sustainable and more useful than both plastic and synthetic material; differences between synthetic material and plastic were smaller and did not reach significance after Sidak correction (see Supplementary Section I). Green-technology-specific benefit information increased PMS, *M*_*diff*_ = 0.41, *SE* = 0.14, 95% CI [0.13, 0.70], and PMU, *M*_*diff*_ = 0.33, *SE* = 0.13, 95% CI [0.08, 0.58]. Interactions were not significant for either outcome. Thus, the predicted polymer–plastic difference in H4 and the information effects in H5 were supported. However, the analyses provided no evidence that green-technology-specific benefit information attenuated term-based differences (RQ1). Conclusions remained the same after controlling for prior green-technology use.

**TABLE 4:** Direct experimental effects (study 2).

Outcome	Effect	*F*	*df*	*p*	partial η^2^
Perceived material sustainability	Material term	37.21	2, 384	< 0.001	0.162
	Information condition	8.27	1, 384	0.004	0.021
	Material term × information condition	0.82	2, 384	0.441	0.004
Perceived material usefulness	Material term	11.19	2, 384	< 0.001	0.055
	Information condition	6.50	1, 384	0.011	0.017
	Material term × information condition	0.20	2, 384	0.816	0.001
Perceived technology sustainability	Material term	1.27	2, 384	0.282	0.007
	Information condition	4.06	1, 384	0.045	0.010
	Material term × information condition	0.80	2, 384	0.452	0.004
Perceived technology usefulness	Material term	1.32	2, 384	0.267	0.007
	Information condition	4.13	1, 384	0.043	0.011
	Material term × information condition	1.32	2, 384	0.267	0.007
Intention to use	Material term	0.34	2, 384	0.713	0.002
	Information condition	1.29	1, 384	0.257	0.003
	Material term × information condition	3.09	2, 384	0.047	0.016

Univariate analyses of technology perceptions largely yielded non-significant effects. Although the univariate information effects on PTS and PTU were nominally significant and small (see Table 4), neither remained significant after controlling for prior green-technology use. For ITU, neither the main effect of material term nor the main effect of information condition was significant. The term × information interaction was significant but small; however, follow-up simple effects showed no significant information effect within any material term condition (see Supplementary Table I.2). The exploratory analyses therefore yielded limited evidence of direct effects on distal outcomes (RQ2).

Sidak-corrected pairwise comparisons, and robustness analyses controlling for prior green-technology use are reported in the Supplementary Section I.

#### 3.2.2 Material-to-technology transfer model

The hypothesised corresponding-path model showed inadequate global fit, χ^2^(2) = 38.98, *p* < 0.001, CFI = 0.969, TLI = 0.568, RMSEA = 0.218, SRMR = 0.058. Model diagnostics indicated a potentially omitted path from PMU to PTS. Because this path had not been specified *a priori*, we treated its addition as a data-informed extension of the hypothesised model, although the path had a plausible interpretation in the green-technology context. Adding this path substantially improved model fit, and the path was therefore retained in the extended model. For the final model, we constrained non-significant direct paths from material term and information condition to technology perceptions and ITU to zero for reasons of parsimony. The final model showed good fit, χ^2^(10) = 13.14, *p* = 0.216, CFI = 0.997, TLI = 0.993, RMSEA = 0.028, SRMR = 0.033, and explained 18.4% of the variance in PMS, 7.3% in PMU, 16.2% in PTS, 21.0% in PTU, and 54.3% in ITU. Because the PMU-to-PTS path was added based on model diagnostics, this final model should be regarded as partly data-informed rather than as a purely confirmatory test of the initially specified path structure. The material-term contrasts and information condition predicted material perceptions, which in turn were associated with technology perceptions (see Figure 1). PMS was associated with PTS, PMU with PTU and also PTS. PTS and PTU were both significantly associated with ITU, with PTU showing the strongest association. Full model comparisons and path coefficients are reported in the Supplementary Section J. Indirect effects were consistent with the proposed material-to-technology transfer process (Table 5). The sustainability pathway yielded small but significant indirect effects. The usefulness pathway was stronger and more consistent, with significant indirect effects for synthetic material versus plastic, polymer versus plastic, polymer versus synthetic material, and green-technology-specific benefit information. The exploratory cross-domain pathway through PMU and PTS was also significant. Total serial indirect effects were significant for all experimental predictors, with the strongest effects observed for polymer. Thus, the observed association pattern was consistent with H6, and the bootstrapped indirect effects were consistent with the pathways proposed in H7. Robustness analyses controlling for prior green-technology use did not change the substantive conclusions and are reported in the Supplementary Section J.

**FIGURE 1:**
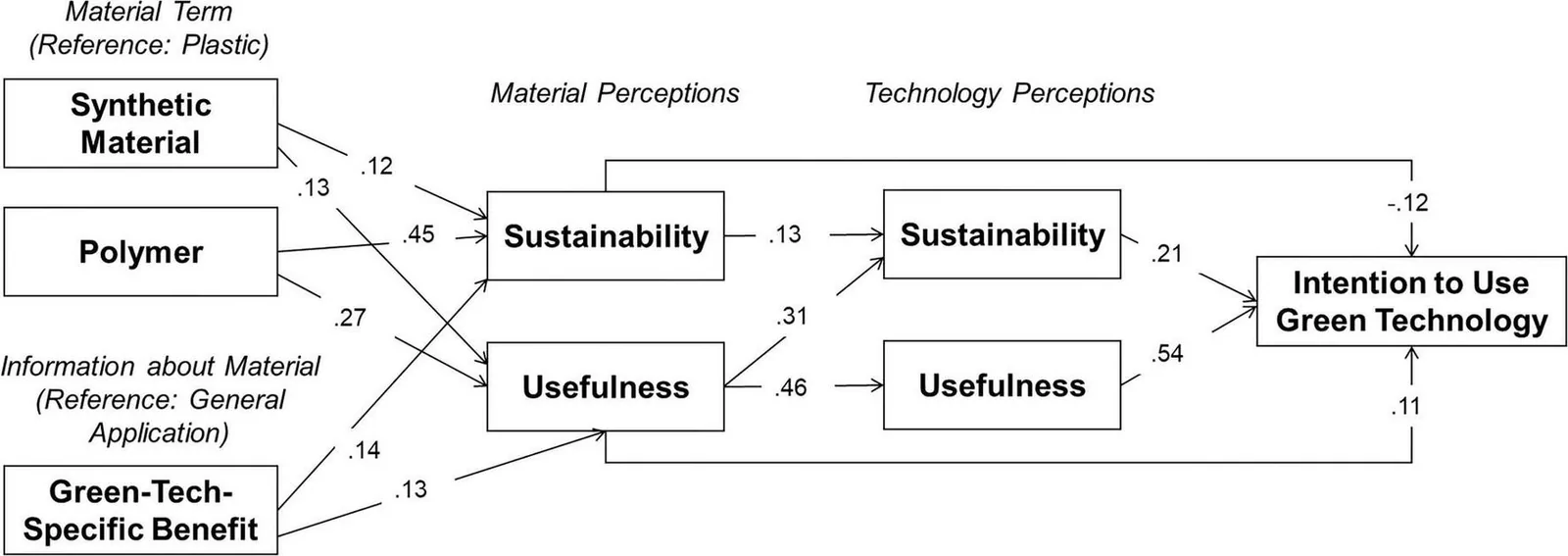
Final path model with standardised effects. All displayed structural paths were statistically significant at *p* < 0.05. Residual covariances between the two material perceptions and between the two technology perceptions are omitted for clarity. Plastic and the general application information condition served as the reference categories.

**TABLE 5:** Bootstrap indirect effects (study 2).

Pathway	Synthetic material vs. plastic	Polymer vs. plastic	Polymer vs. synthetic material	Green-technology-specific benefit information
Sustainability pathway	0.015 [0.002, 0.048]	0.056 [0.014, 0.136]	0.041 [0.010, 0.103]	0.016 [0.003, 0.050]
Usefulness pathway	0.144 [0.021, 0.332]	0.309 [0.170, 0.544]	0.166 [0.061, 0.333]	0.137 [0.039, 0.295]
Cross-domain pathway	0.038 [0.006, 0.126]	0.082 [0.022, 0.206]	0.044 [0.008, 0.133]	0.036 [0.007, 0.112]
Total serial indirect effect	0.197 [0.033, 0.402]	0.447 [0.280, 0.660]	0.251 [0.096, 0.424]	0.189 [0.054, 0.359]

Values are unstandardised indirect effects with 95% bias-corrected bootstrap confidence intervals in brackets. Confidence intervals were based on 5,000 bootstrap samples. The sustainability pathway refers to the indirect effect through perceived material sustainability → perceived technology sustainability → intention to use. The usefulness pathway refers to the indirect effect through perceived material usefulness → perceived technology usefulness → intention to use. The cross-domain pathway refers to the indirect effect through perceived material usefulness → perceived technology sustainability → intention to use. The total serial indirect effect represents the sum of these serial indirect pathways.

## 4 Discussion

Across two experimental survey studies, material terminology shaped multidimensional material meanings, with the clearest effects at the proximal material level. Study 1 showed that plastic elicited less favourable attitudes toward material use than synthetic material and polymer and activated less favourable ecological and functional-material evaluations, and less favourable safety-related material impression, whereas polymer was markedly less familiar. Ecological and functional-material meanings provided the most robust statistical pathways linking terminology with attitudes toward material use. Study 2 again showed clear terminology effects on perceived material sustainability and usefulness and showed that green-technology-specific benefit information additionally increased these perceptions but did not attenuate terminology-based differences. Evidence at the technology level was more bounded. Direct experimental effects on technology perceptions and ITU were weak or absent, but material perceptions were associated with technology perceptions through corresponding sustainability and usefulness pathways. The usefulness pathway was particularly pronounced, and the data-informed PMU to PTS path suggested that functional material evaluations may also contribute to sustainability-related technology judgements. Thus, the studies indicate that material terminology and green-technology-specific benefit information can shape proximal material meanings, while downstream relevance for technology evaluation is indirect and should be interpreted as associational rather than as a fully established causal sequence.

### 4.1 Material terminology and multidimensional material meanings

The term-dependent profiles observed across the two studies indicate that plastic, synthetic material, and polymer were not psychologically interchangeable in lay judgement. Importantly, these differences should not be understood as pure wording effects on an identical material, because the three terms refer to related but not technically equivalent material categories. Rather, the terms are likely to evoke somewhat different lay representations of the material. Consistent with the free-association findings, plastic more readily brought to mind packaging, waste, and environmental harm, whereas polymer more often elicited science- and chemistry-related associations. Thus, the observed effects reflect both the terminology used and the meanings and category representations associated with that terminology. A stricter test of pure relabelling would require presenting the same specific material or component with identical physicochemical properties under different labels.

This interpretation is consistent with framing and cue-utilisation approaches, which propose that the way an object is described can make particular aspects of it more accessible in judgement (Entman, 1993; Tversky and Kahneman, 1981). Previous research has shown such effects for sustainability claims, naturalness cues, eco-labels, and packaging information (Schuldt and Schwarz, 2010; Siegrist and Sütterlin, 2017; Sörqvist et al., 2015; Steenis et al., 2017). The present findings suggest that similar processes may already operate at the level of the basic material term itself.

This matters particularly for plastics and related polymer-based materials because they are associated with both environmental problems and substantial functional benefits. Plastics are implicated in waste, pollution, persistence, and ecological harm (Borrelle et al., 2020; Geyer et al., 2017; MacLeod et al., 2021), while their material properties also support applications in medicine, transport, construction, packaging, and energy-related technologies (Andrady and Neal, 2009). Public perceptions similarly combine concern about environmental consequences with recognition of convenience, versatility, affordability, hygiene, practicality, and usefulness (Heidbreder et al., 2019). The present findings suggest that terminology changes the relative prominence of these meanings. Plastic was more strongly linked to environmental harm and negative evaluation; polymer was linked more strongly to technical and unfamiliarity-related meanings; and synthetic material generally occupied a less negative position than plastic, with an especially clear intermediate pattern in Study 2.

A key implication is that material meanings were not unidimensional. The terms did not simply make the material appear more or less positive overall; they produced differentiated profiles across ecological, functional-material, safety-related, and familiarity-related perceptions. This aligns with attitude-network research showing that attitudes toward conventional and bio-based plastics consist of multiple evaluative reactions, including beliefs, emotions, and behavioural tendencies (Zwicker et al., 2020). It also converges with research on plastic medical devices showing that material preferences are embedded in broader networks of environmental beliefs, safety beliefs, emotions, and trust (Chambon et al., 2023). The present findings add that such material-related meanings are not only relevant to plastic evaluations in general, but can also be differentially activated by the terminology used to describe the material.

Ecological and functional meanings emerged as the most robust explanatory dimensions. This is theoretically consistent with prior work showing that plastic-related attitudes combine environmental concern with perceived usefulness rather than reflecting a uniformly negative evaluation (Heidbreder et al., 2019; Zwicker et al., 2020). In Study 1, ecological value and functional-material value statistically accounted for terminology differences in attitudes toward material use and remained robust when broader evaluative impressions and additional semantic-differential facets were included. These findings suggest that material terminology matters because it can shift which aspects of polymer-based materials become salient: their ecological implications or their functional-material value.

Familiarity followed a different logic. Polymer was clearly less familiar than plastic and synthetic material, which is consistent with its more technical semantic register and its scientific meaning as a macromolecular material category (Jenkins et al., 1996). However, familiarity did not explain attitudes toward material use once ecological, functional-material, and safety-related meanings were considered. This pattern is consistent with research on unfamiliar technologies showing that names can shape emerging attitudes by activating associations with more familiar categories (Boersma et al., 2019), and with research on nanotechnology showing that familiarity, risk perceptions, benefit perceptions, and acceptance are closely connected (Capon et al., 2015; Siegrist and Keller, 2011). Thus, unfamiliarity appeared to mark the technicality of the term polymer, but it did not by itself undermine attitudes toward material use in Study 1. More technical material terminology can therefore be evaluated favourably despite lower familiarity, although it may still require contextual explanation to remain transparent and understandable.

Safety-related material impression requires a more cautious interpretation. Such meanings are theoretically plausible because lay evaluations of materials and technologies often involve risk, trust, affect, and perceived contamination, particularly when material composition, production processes, or downstream consequences are difficult to observe directly (Finucane et al., 2000; Huijts et al., 2012; Siegrist and Cvetkovich, 2000; Slovic, 1987). For plastics specifically, research on products made from used recycled plastic bottles suggests that such materials can evoke contact-related contamination concerns, particularly when the resulting product was worn against the skin (Meng and Leary, 2021). This concerns perceived contamination during product use rather than ecological contamination across the material life cycle. In the present data, safety-related material impression accounted for terminology differences in the mediation model, but this pathway was less robust than the ecological and functional-material pathways: when overall evaluation was included, safety-related material impression no longer explained unique indirect effects. This pattern suggests that safety, cleanliness, and trustworthiness were partly embedded in a broader affective-evaluative material impression. The exploratory free associations further illustrate the heterogeneous nature of this domain. Spontaneous safety-related meanings were distributed across distinct categories referring to human-health risks, general danger and trust, health or hygiene, and environmental forms of harmfulness. Thus, the free-association findings do not point to a single, coherent lay conception of “material safety,” but rather to several distinct meanings that may be combined in broad evaluative ratings. Future research should therefore assess distinct safety domains separately, including personal or health-related safety during use, technical or product safety, and environmental safety across production, use, and end-of-life stages.

The exploratory free-association findings provided supplementary triangulation for the rating-based results. Whereas the semantic differential assessed predefined material-meaning dimensions derived from prior research and expert input, the free-association task captured spontaneously accessible meanings in participants’ own words before they completed the rating scales. The independently coded responses broadly converged with the semantic-differential findings: plastic was more strongly associated with ecological harm and unspecified negative evaluations, whereas polymer more frequently elicited science- and chemistry-related meanings and unfamiliarity. Synthetic material generally showed a less polarised pattern across these focal categories. The principal contrasts were also retained when the analyses were restricted to participants’ first association, reducing concern that they merely reflected condition differences in the number of responses provided. These findings strengthen the interpretation that the terminology conditions activated distinct lay representations of the material rather than producing differences limited to predefined rating scales. Because the analysis was exploratory and involved multiple category comparisons, however, the findings should be interpreted as supplementary convergent evidence rather than as independent confirmatory tests.

### 4.2 Material-to-technology transfer of meaning

Study 2 examined whether material meanings remain confined to the material itself or are linked to evaluations of green technologies in which such materials are used. This question builds on research showing that public evaluations of sustainable energy technologies depend not only on technical performance or environmental benefit, but also on perceived benefits, risks, costs, trust, fairness, affect, and contextual meanings (Batel, 2020; Huijts et al., 2012; Perlaviciute and Steg, 2014). The present findings add a material-terminology perspective to this literature: terminology- and information-based differences in material perceptions were relevant for technology evaluations through indirect transfer pathways, even though direct experimental effects on technology perceptions and ITU were weak or absent.

Consistent with this transfer logic, PMS and PMU were associated with corresponding technology perceptions, which in turn were associated with ITU. However, the pathways differed in strength: the usefulness pathway was substantially stronger than the sustainability pathway. This pattern is consistent with technology acceptance research, in which perceived usefulness and performance expectancy are central predictors of behavioural intention (Davis, 1989; Venkatesh et al., 2003), and with research on renewable and low-carbon technology adoption showing that perceived benefits are among the strongest predictors of adoption intentions (Scheller et al., 2024; Schulte et al., 2022).

The additional path from PMU to PTS suggests that functional and sustainability-related meanings were not fully independent in the green-technology context. This path was not part of the strictly corresponding hypothesised model and should therefore be interpreted as a data-informed, theoretically plausible extension. One possible explanation is that materials perceived as functionally enabling, durable, and efficient also made the technologies in which they were used appear more viable and sustainable. This interpretation aligns with evidence that sustainability and functional quality can become intertwined in product and technology evaluations and that durability and efficiency are central to resource-efficiency reasoning (Milios and Dalhammar, 2023; Skard et al., 2021). It nevertheless remains a *post hoc* finding that requires direct testing.

At the same time, the evidence points to a bounded form of material-to-technology transfer. The experimental manipulations directly targeted the material, and their strongest and most reliable effects were observed for proximal material perceptions. This pattern is consistent with the principle of compatibility, according to which predictors and outcomes are more closely related when they correspond in target and specificity (Ajzen, 2020). It also fits research on sustainable energy and low-carbon technology acceptance showing that technology perceptions and use intentions are shaped by broader constellations of perceived benefits, risks, costs, trust, norms, and perceived control (Huijts et al., 2012; Scheller et al., 2024; Schulte et al., 2022). The indirect-effect pattern therefore supports the proposed transfer logic, but it should not be interpreted as definitive causal evidence for the full mediator-to-outcome sequence.

One additional finding warrants caution. In the final path model, PMS had a small negative residual association with ITU after PTS, PTU, PMU, and the experimental predictors were taken into account. This coefficient should not be interpreted as evidence that perceiving a material as sustainable reduces intention to use green technologies. It is more plausibly treated as a residual or suppression effect arising in a model with strongly related material and technology evaluations (MacKinnon et al., 2000; Watson et al., 2013). Because the substantive meaning of this residual variance is unclear (e.g., ambivalent or sceptical material meanings, or trade-offs with perceived functionality or quality, Skard et al., 2021), future research is needed to examine this in more detail.

### 4.3 Information effects and communication implications

The information manipulation showed that material evaluations were not determined by terminology alone. Green-technology-specific benefit information increased PMS and PMU relative to general application information. Importantly, this effect should be interpreted as an effect of the specific positively valenced functional and sustainability-related content that was provided, rather than as evidence that additional context *per se* improves material evaluations. Furthermore, it did not eliminate term-based differences. Thus, material terminology and green-technology-specific benefit information appeared to operate as distinct cues: terminology shaped the meanings associated with the material, whereas the information condition made favourable application-specific functional and sustainability-related contributions salient.

This pattern has implications for sustainability communication. The findings do not imply that one material term is generally preferable to another. Rather, they suggest that material terms should be used transparently and explained in relation to the application in which the material is used. Although polymer elicited more favourable material perceptions than plastic, it was also less familiar; and although familiarity did not explain attitudes toward material use in Study 1, unfamiliar terminology may still require explanation to remain understandable. Conversely, more familiar terms may carry stronger everyday associations with waste, disposability, or pollution. Effective communication should therefore make clear which material is meant, why it is used in a specific application, which functional properties it contributes, and which environmental benefits and trade-offs are involved.

This is particularly important because the environmental meaning of polymer-based materials depends strongly on application context. Life-cycle and circular-economy perspectives similarly emphasise that environmental implications depend on production, use context, functional performance, lifetime, and end-of-life pathways rather than on material category alone (Jiao et al., 2024; Nordahl and Scown, 2024; Pellengahr et al., 2023). The same broad material category may be environmentally problematic in disposable applications but functionally relevant in long-lived technologies that require durability, insulation, weather resistance, or lightweight components.

The findings also raise the possibility of what might be termed “terminological green framing”: the use of material terminology that elicits more favourable environmental inferences without itself providing evidence of better environmental performance. In the present studies, the more technical term polymer was associated with more favourable environmental perceptions than plastic, illustrating how such a process might arise. This possibility connects to the broader literature on greenwashing and environmental claims, which shows that environmental impressions can be shaped by explicit as well as implicit or insufficiently substantiated environmental cues (de Freitas Netto et al., 2020; Schmuck et al., 2018). Importantly, the present studies do not demonstrate such a pure relabelling effect for an objectively identical component, because the concrete material referent remained unspecified and the terms denote partly overlapping rather than technically identical categories. Regardless, the communication goal should not be to avoid negative associations with plastic or to make contested materials appear more sustainable through technical terminology. Rather, the findings point to the need for transparent, context-sensitive communication that avoids both simplified “plastic is bad” narratives and unsubstantiated “polymer is sustainable” claims. This aligns with broader guidance on environmental claims, which stresses that such claims should be specific, substantiated, and not misleading (OECD, 2025). Public communication about sustainability transitions should support differentiated material judgements by connecting material terms to concrete applications, functional roles, and environmental consequences.

### 4.4 Limitations and future research

Several limitations should be considered. First, the studies were conducted in German and focused on the terms Plastik, Kunststoff, and Polymer. The English renderings plastic, synthetic material, and polymer are useful approximations, but they should not be treated as exact translation equivalents. The semantic distinctions between the original terms are embedded in the German linguistic context, and corresponding terms in other languages may differ in familiarity, technicality, affective connotation, and association with waste or pollution. This limitation is consistent with broader concerns in cross-cultural research that translation requires semantic and conceptual equivalence rather than literal word matching (Brislin, 1970; van de Vijver and Leung, 1997). Future research should therefore examine whether similar terminology effects emerge in other linguistic and cultural contexts.

Second, the terms refer to related but not technically identical material categories. The specific material referent was intentionally left unspecified because the study focused on non-expert communication, in which category boundaries may not be represented in the same way as in materials science. Nevertheless, technical distinctions matter. Polymer is a broader scientific category than plastic, whereas plastic refers to a polymeric material rather than serving as a technical synonym for polymer. The German terms Plastik and Kunststoff likewise overlap in everyday use but are not fully interchangeable in all contexts. Moreover, because no specific physicochemical material was held constant across terminology conditions, the observed effects cannot be interpreted as pure wording effects on the evaluation of an identical material. They reflect the combined consequences of material terminology and the category representations evoked by those terms in lay communication. A particularly informative next step could therefore be to hold a specific component and its physicochemical properties constant and manipulate only whether it is described, for example, as a plastic component or a polymer component. Such research could test more directly whether terminology alone can create specific inferences in the absence of material change.

Third, the mediation and path models were based on post-manipulation measures collected in the same survey context. The experimental manipulations support causal conclusions about the effects on material perceptions. However, associations among material perceptions, technology perceptions, and ITU are correlational. The indirect effects are therefore consistent with the proposed meaning-transfer process but do not establish the causal order of mediators and outcomes. This is particularly important because cross-sectional mediator-outcome data can yield biased estimates of longitudinal or causal processes (Imai et al., 2010; Maxwell and Cole, 2007). Experimental-causal-chain approaches or temporally separated designs are needed to test whether changes in material perceptions causally precede changes in technology perceptions and intentions (Spencer et al., 2005). In addition, the final path structure was partly data-informed: the PMU-to-PTS path was added after inspection of model diagnostics, and non-significant direct paths were subsequently constrained to zero. The final model should therefore be regarded as a plausible explanatory model requiring independent replication rather than as a purely confirmatory test of the complete proposed causal structure.

Fourth, Study 2 used a generic green-technology context, and the material-level measures were application-specific rather than entirely context-free. PMU explicitly referred to the material’s usefulness in green technologies. This increased correspondence with the manipulation and proposed process but also increased conceptual proximity between material and technology perceptions. Moreover, participants evaluated green technologies in general rather than a specific technology such as solar panels, wind turbines, or batteries. Future research should separate context-free from application-specific material evaluations and compare concrete technologies in which polymer-based materials serve different functional and environmental roles.

Fifth, the results need to be interpreted in light of the specific design of the information manipulation used in Study 2. The benefit-focused condition highlighted favourable functional properties and explicitly linked them to safe and efficient long-term functioning and sustainable resource use, whereas the comparison condition provided more neutral information about forms and applications. Thus, the observed information effects may reflect application specificity, positive valence, functional-benefit information, sustainability-related framing, or a combination of these features; the present design cannot disentangle these components. Accordingly, the findings should not be generalised to all forms of material information. Future studies should compare different types of information, such as purely technical information, balanced trade-off information, life-cycle information, or messages explicitly acknowledging environmental concerns. Such work would help identify which forms of information support more differentiated and context-sensitive material evaluations.

Finally, the studies have limitations regarding sample generalisability and the behavioural scope of the outcomes. Online adult panels are appropriate for studying lay perceptions, but the samples were not designed to represent all population segments, stakeholder groups, or expert communities. In addition, the primary outcomes were self-reported attitudes, perceptions, and intentions rather than behavioural choices or real-world acceptance decisions. This is important because intentions do not necessarily translate into behaviour, and intention–behaviour gaps are well documented (Sheeran, 2002). Future research should therefore examine whether material terms influence actual choices, support for technology deployment, willingness to accept infrastructure, or policy preferences, and should compare lay audiences with experts, practitioners, and stakeholders involved in sustainability communication or technology implementation.

### 4.5 Conclusion

Across two studies, material terminology elicited different lay evaluations and material meanings for technically non-equivalent but partly overlapping material categories. In Study 1, participants reported less favourable attitudes toward material use when the material was described as plastic rather than synthetic material or polymer, and these differences were statistically accounted for especially by ecological and functional-material evaluations. These effects should be understood as terminology- and category-representation effects rather than as evidence that an objectively identical material changes meaning solely through relabelling. In Study 2, terminology again affected proximal material perceptions, while green-technology-specific benefit information additionally increased perceived material sustainability and usefulness without eliminating terminology effects.

Material perceptions were associated with green-technology evaluations through indirect pathways, particularly perceived usefulness, although these downstream associations do not establish a causal mediator-to-outcome sequence. More broadly, the findings concern perceived sustainability and material meaning rather than objective environmental performance. They also highlight a potential risk of terminological green framing when technical material terms generate favourable environmental inferences without corresponding evidence about material composition or life-cycle impacts. Communication about contested materials should therefore use terminology transparently and connect material names to specific applications, functional roles, environmental trade-offs, and substantiated environmental evidence.

## Data Availability

The raw data supporting the conclusions of this article will be made available by the authors, without undue reservation.
