# Supplementary material for: Plastic, synthetic material, or polymer? How material terminology shapes material meanings and informs green technology evaluations
Source: Front Psychol. 2026 Sep 22;17:1951034. doi: 10.3389/fpsyg.2026.1951034 (PMC13638414; doi:10.3389/fpsyg.2026.1951034)
Supplement: Supplementary file 1 [file Supplementary_file_1.pdf]

Supplementary Material

A. Sample Size Planning 2

B. Data-Quality Screening 3

C. Study 1 Semantic Differential 4

D. Study 1: Post-Hoc Comparisons 6

E. Study 1: Robustness Analyses 7

F. Study 1: Exploratory Analysis of Initial Free Associations 9

G. Study 2: Manipulation Materials and Checks 23

H. Study 2: Measurement Model 26

I. Study 2: Direct Experimental Effects 28

J. Study 2: Path Model 32

A. Sample Size Planning

A priori power analyses were conducted before data collection using G*Power 3.1 (Faul et al., 2009). Sample-size planning focused on the between-subjects experimental effects. For both studies, the analyses assumed a significance level of $\alpha = .05$, statistical power of 80%, and a small-to-medium effect size of $f = .18$.

For Study 1, the analysis was based on a one-way between-subjects ANOVA with three material-terminology conditions and indicated a required total sample size of $N = 301$. Recruitment exceeded this minimum to allow for exclusions resulting from the predefined data-quality screening criteria.

For Study 2, the analysis was based on the focal effects in the 3 × 2 between-subjects factorial design. The calculation was specified for an effect with two numerator degrees of freedom, corresponding to the three-level material-terminology main effect or the material terminology × information interaction, and indicated a required total sample size of $N = 301$. Again, recruitment exceeded this minimum to accommodate anticipated exclusions resulting from the general and manipulation-specific data-quality screening criteria and to increase the precision of the estimated experimental effects.

<u>Reference:</u>

Faul, F., Erdfelder, E., Buchner, A., and Lang, A.-G. (2009). Statistical power analyses using G*Power 3.1: tests for correlation and regression analyses. *Behav. Res. Methods* 41, 1149–1160. doi: 10.3758/BRM.41.4.1149

B. Data-Quality Screening

Responses were screened for insufficient data quality using predefined criteria based on recommendations for identifying careless and low-quality responses in online survey data (Curran, 2016; DeSimone et al., 2015; Meade and Craig, 2012; Zhang and Conrad, 2014). Participants were flagged for a high proportion of missing values if at least 20% of their responses were missing. Extremely short completion time was flagged if total survey duration was less than one third of the median completion time. Page-level response quality was assessed for pages with at least five items using the same response format. Pages were flagged when responses indicated near-zero response variability or unrealistically short page completion time. This page-level indicator contributed to the overall exclusion decision only when at least two of four eligible pages were flagged.

In Study 1, open-ended responses were also screened. Participants received a strict open-response flag when the free-association responses were missing or consisted entirely of nonsensical content, and a mild open-response flag when responses were partly missing or partly nonsensical. Participants were excluded immediately if they received a strict open-response flag. They were also excluded if they received a mild open-response flag in combination with at least one additional quality flag, or if they received at least two other quality flags.

In Study 2, we additionally screened responses for manipulation-specific quality indicators. Responses to the open material manipulation check were coded as a strict exclusion flag if participants gave no answer, a completely irrelevant answer, or a nonsensical answer; vague or partially incorrect responses were coded as a loose flag. Reading time for the manipulation text was coded as a strict exclusion flag if participants spent less than 5 seconds on the text, and as a loose flag if they spent between 5 and 10 seconds on the text. Participants were excluded if they met any strict exclusion criterion. In addition, participants were excluded if they had both loose manipulation-related flags, one loose manipulation-related flag combined with at least one additional data-quality flag, or at least two other data-quality flags.

References:

Curran, P.G. (2016). Methods for the detection of carelessly invalid responses in survey data. *J. Exp. Soc. Psychol. 66*, 4–19. doi: 10.1016/j.jesp.2015.07.006

DeSimone, J.A., Harms, P.D., and DeSimone, A.J. (2015). Best practice recommendations for data screening. *J. Organ. Behav. 36*, 171–181. doi: 10.1002/job.1962

Meade, A.W., and Craig, S.B. (2012). Identifying careless responses in survey data. *Psychol. Methods 17*, 437–455. doi: 10.1037/a0028085

Zhang, C., and Conrad, F. G. (2014). Speeding in web surveys: the tendency to answer very fast and its association with straightlining. *Surv. Res. Methods 8*, 127–135. doi: 10.18148/srm/2014.v8i2.5453

C. Study 1 Semantic Differential

Material perceptions were assessed using a semantic differential. The item pool was developed in two steps. First, we derived core material-perception domains from prior work showing that plastic-related attitudes include environmental concerns, perceived usefulness, perceived risks, and affective associations (Heidbreder et al., 2019; Zwicker et al., 2020). This literature-informed part of the item pool covered ecological and normative evaluations, functional-material evaluations, safety-related evaluations, and additional material-related associations relevant to plastic perception. Second, the item pool was refined in collaboration with experts in polymer and materials science. This interdisciplinary refinement led us to retain or add adjective pairs that were considered particularly relevant for comparing the material terms plastic, synthetic material, and polymer, including innovativeness, complexity, and familiarity. Finally, two broad evaluative adjective pairs were included to capture general evaluative impressions. Overall, participants evaluated the assigned material term on 15 bipolar adjective pairs using 5-point scales.

For the focal analyses, semantically related adjective pairs were averaged into theory-driven composite indices. Ecological value comprised the adjective pairs *not sustainable–sustainable*, *environmentally harmful–environmentally friendly*, and *irresponsible–responsible*. Functional-material value comprised *useless–useful*, *low value–high value*, and *outdated–innovative*. Safety-related material impression comprised *dangerous–safe*, *not trustworthy–trustworthy, dirty–clean.* Familiarity was analysed with the single item *unfamiliar–familiar*.

Several adjective pairs were not included in the main aggregated dimensions but were retained for robustness analyses. *Overall evaluation*, based on the adjective pairs *bad–good* and *unattractive–attractive* (α = .73), was excluded from the main model because it captures a broad affective-evaluative impression that is conceptually close to the outcome, attitude toward material use. It was therefore included only in a robustness analysis. Naturalness (*artificial–natural*) was retained as a robustness facet because it reflects a genuine material-related perception, but it was not included in ecological value: In the context of synthetic or polymeric materials, perceived naturalness may reflect a naturalness heuristic rather than environmental sustainability per se. Longevity (*short-lived–long-lasting*) was also retained as a robustness facet because it is materially central but evaluatively ambivalent. Being long-lasting can be positive in terms of durability and functional performance, but it can also be negative in environmental contexts where persistence and slow degradation are problematic. Finally, complexity (*complex–simple)* was included because the term *polymer* may activate a more technical and complex understanding of the material, an issue that was also considered relevant by the polymer/materials-science members of the project team.

Figure C.1 displays the mean item-level semantic-differential ratings by material-term condition. In line with the composite-level analyses reported in the main text, item-level analyses showed that the three terms elicited distinct semantic profiles.

References:

Heidbreder, L.M., Bablok, I., Drews, S., and Menzel, C. (2019). Tackling the plastic problem: a review on perceptions, behaviors, and interventions. *Sci. Total Environ. 668*, 1077–1093. doi: 10.1016/j.scitotenv.2019.02.437

Zwicker, M.V., Nohlen, H.U., Dalege, J., Gruter, G.-J.M., and van Harreveld, F. (2020). Applying an attitude network approach to consumer behaviour towards plastic. *J. Environ. Psychol. 69*:101433. doi: 10.1016/j.jenvp.2020.101433

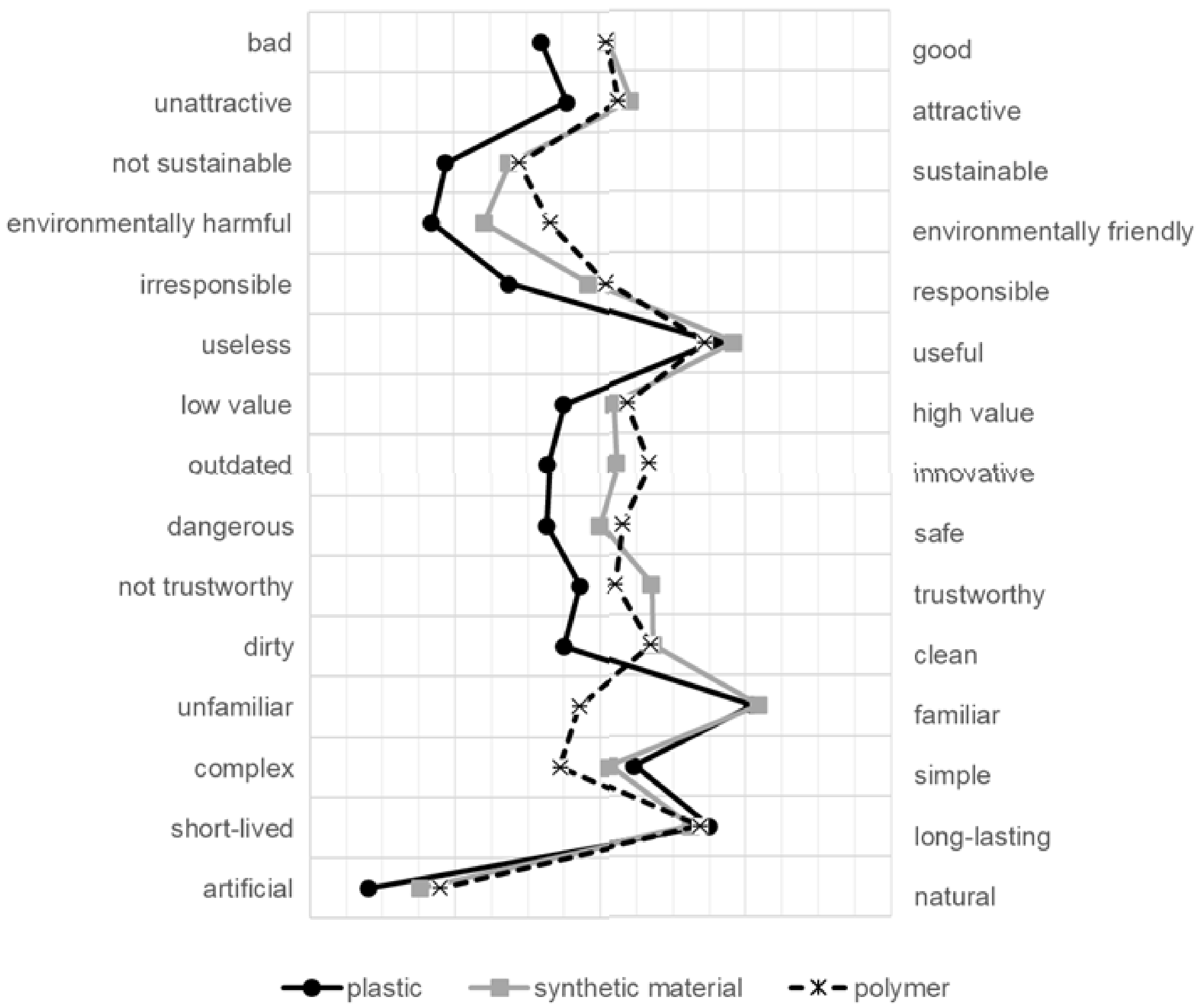

Figure C.1. Mean Semantic Differential Ratings by Material Term (Study 1).

D. Study 1: Post-Hoc Comparisons

Table D.1. Games–Howell Pairwise Comparisons for Study 1 Outcomes.

Variable	Comparison	*Mean difference*	*SE*	*p*	95% CI
Attitude toward material use	Plastic – Synthetic material	-0.66	0.19	.002	[-1.11, -0.21]
	Plastic – Polymer	-0.51	0.19	.024	[-0.96, -0.05]
	Synthetic material – Polymer	0.15	0.18	.665	[-0.27, 0.57]
Ecological value	Plastic – Synthetic material	-0.45	0.14	.005	[-0.78, -0.12]
	Plastic – Polymer	-0.66	0.13	< .001	[-0.98, -0.35]
	Synthetic material – Polymer	-0.22	0.14	.248	[-0.54, 0.10]
Functional-material value	Plastic – Synthetic material	-0.32	0.11	.013	[-0.59, -0.06]
	Plastic – Polymer	-0.35	0.12	.009	[-0.62, -0.07]
	Synthetic material – Polymer	-0.03	0.11	.972	[-0.29, 0.24]
Safety-related material impression	Plastic – Synthetic material	-0.49	0.13	< .001	[-0.79, -0.19]
	Plastic – Polymer	-0.46	0.13	.002	[-0.77, -0.15]
	Synthetic material – Polymer	0.03	0.12	.965	[-0.26, 0.32]
Familiarity	Plastic – Synthetic material	-0.05	0.14	.936	[-0.37, 0.27]
	Plastic – Polymer	1.19	0.15	< .001	[0.83, 1.56]
	Synthetic material – Polymer	1.24	0.15	< .001	[0.89, 1.59]

Note. Positive values indicate higher scores in the first-named condition. Semantic-differential dimensions were assessed on 5-point scales; attitude toward material use was assessed on a 7-point scale.

E. Study 1: Robustness Analyses

We conducted two robustness analyses to examine whether the mediation findings were sensitive to broader evaluative impressions and additional semantic-differential facets.

First, the mediation model reported in the main text was repeated including overall evaluation as an additional mediator. Overall evaluation was based on the two broad evaluative semantic-differential items *bad–good* and *unattractive–attractive*. It was not included in the focal mediation model because it captures a broad affective-evaluative impression and is conceptually close to attitude toward material use. The model explained 60.8% of the variance in attitude toward material use, $R^2 = .608$, $F(7, 295) = 65.29$, $p < .001$. Overall evaluation was strongly related to attitude toward material use, $B = 0.59$, $SE = 0.09$, $p < .001$. Ecological value remained a significant predictor, $B = 0.28$, $SE = 0.07$, $p < .001$, as did functional-material value, $B = 0.25$, $SE = 0.09$, $p = .007$. Safety-related material impression was no longer significant when overall evaluation was included, $B = 0.15$, $SE = 0.09$, $p = .088$. Familiarity remained non-significant, $B = 0.00$, $SE = 0.05$, $p = .999$.

The indirect effects through overall evaluation were significant for both contrasts. Synthetic material, relative to plastic, had a significant indirect effect on attitude toward material use through overall evaluation, indirect effect = 0.26, $SE = 0.09$, 95% CI [0.11, 0.48]. Polymer, relative to plastic, also had a significant indirect effect through overall evaluation, indirect effect = 0.23, $SE = 0.09$, 95% CI [0.08, 0.44]. Indirect effects through ecological value and functional-material value remained significant for both contrasts. By contrast, the indirect effects through Safety-related material impression were no longer significant when overall evaluation was included: synthetic material versus plastic, indirect effect = 0.08, $SE = 0.05$, 95% CI [-0.01, 0.20]; polymer versus plastic, indirect effect = 0.07, $SE = 0.05$, 95% CI [-0.01, 0.20]. Indirect effects through familiarity remained non-significant. Thus, including overall evaluation did not alter the substantive conclusion that terminology effects on attitudes toward material use were statistically accounted for by material perceptions, but it showed that overall evaluation absorbed part of the shared affective-evaluative variance, especially variance shared with safety-related material impression.

Second, we estimated an expanded robustness model including overall evaluation and additional semantic-differential facets that were not part of the composite dimensions. Specifically, the model included ecological value, functional-material value, safety-related material impression, familiarity, overall evaluation, naturalness, longevity, and complexity as parallel mediators. The expanded model explained 60.5% of the variance in attitude toward material use, $R^2 = .605$, $F(10, 291) = 44.53$, $p < .001$. Ecological value remained a significant positive predictor, $B = 0.31$, $SE = 0.08$, $p < .001$, as did functional-material value, $B = 0.27$, $SE = 0.10$, $p = .005$, and overall evaluation, $B = 0.58$, $SE = 0.10$, $p < .001$. Safety-related material impression was not significant, $B = 0.14$, $SE = 0.09$, $p = .142$. Familiarity was also not significant, $B = -0.00$, $SE = 0.05$, $p = .995$. None of the additional semantic-differential facets significantly related to attitude toward material use: naturalness, $B = -0.04$, $SE = 0.06$, $p = .500$; longevity, $B = -0.05$, $SE = 0.04$, $p = .274$; and complexity, $B = -0.02$, $SE = 0.05$, $p = .693$.

In the expanded model, significant indirect effects again emerged through ecological value, functional-material value, and overall evaluation for both terminology contrasts. The indirect effects through safety-related material impression were not significant once overall evaluation and the additional facets were included: synthetic material versus plastic, indirect effect = 0.07, $SE = 0.06$, 95% CI [-0.03, 0.20]; polymer versus plastic, indirect effect = 0.06, $SE = 0.05$, 95% CI [-0.02, 0.18]. Indirect effects through familiarity, naturalness, longevity, and complexity were

also non-significant. These findings indicate that the main conclusions were robust to the inclusion of broader evaluative impressions and additional material-related semantic facets. Ecological value and functional-material value remained the most consistent material-perception pathways, whereas safety-related material impression contributed in the main model but overlapped substantially with overall evaluation.

F. Study 1: Exploratory Analysis of Initial Free Associations

F.1. Method

F.1.1. Coding

The coding framework was developed using a deductive–inductive approach. Initial categories reflected theoretically relevant material meanings, including environmental, functional, material-property, technical, application-related, health- and safety-related, familiarity-related, evaluative, and terminological meanings. These categories were iteratively refined, differentiated, or combined based on the observed responses. ChatGPT (GPT-5.6 Thinking; OpenAI) was used as a supporting tool during the exploratory development and refinement of the coding framework. Specifically, it assisted with preliminary semantic grouping, the identification of potentially overlapping categories, and the formulation of possible category labels and boundaries. All suggestions were critically evaluated by the authors. The final framework contained 29 mutually exclusive association-level codes organised into 11 higher-order domains. Definitions, inclusion rules, and verbatim anchor examples are provided in Table F.1.

Each non-empty association field was treated as a separate coding unit. Each coding unit was assigned to exactly one category. Thus, categories were mutually exclusive at the association level. Two coders independently coded all 863 valid associations using the final coding framework. Exact agreement was 94% and Cohen's κ =.936. Disagreements were subsequently resolved by consensus, and the reconciled assignments were used in all analyses.

F.1.2. Participant-Level Aggregation and Statistical Analyses

For the primary analyses, consensus codes were aggregated into binary participant-level indicators. Each indicator represented whether a participant had provided at least one association belonging to the respective category, irrespective of the number of associations within that category. Percentages therefore refer to the proportion of participants in each material-term condition who mentioned the respective category at least once.

Differences between the three material-term conditions were examined using 3 × 2 contingency tables. Pearson chi-square tests were used when expected cell frequencies were adequate; two-sided Fisher–Freeman–Halton exact tests were used for sparse tables. The residual OTHER category was reported descriptively but excluded from inferential testing. Benjamini–Hochberg correction was applied across the 28 omnibus category tests to control the false discovery rate (Benjamini and Hochberg, 1995). Categories that remained significant after this correction were followed up using two-sided Fisher exact tests for the three pairwise condition comparisons, with Holm correction applied within each category. Cramér's V was used as the effect-size measure.

As a sensitivity analysis addressing differences in the number of associations provided, the category comparisons were repeated using only each participant's first association. This gave each participant equal opportunity to contribute one category and captured the most immediately accessible association.

Table F.1. Coding Scheme for Free Associations.

Higher-order category	Code	Category label	Definition and inclusion rule	Anchor examples (German [English])
Environmental meanings	ECO	General environmental and sustainability references	General references to the environment, ecology, or sustainability without an explicitly negative evaluation, a specific harmful consequence, or an explicit reference to recycling, reuse, or circularity.	“Umwelt” [environment]; “Ozean” [ocean]; “umweltfreundlich?” [environmentally friendly?]
	HARM	Negative environmental meanings	Explicitly negative environmental or sustainability-related meanings, including unsustainability, environmental harm, pollution, waste as refuse or litter, environmental persistence, microplastics, and harm to animals or ecosystems. Explicit references to recycling, recyclability, reuse, or single use are coded as CIRCULAR.	“Nicht nachhaltig” [not sustainable]; “Müll” [waste]; “Mikroplastik” [microplastics]; “Umweltverschmutzung” [environmental pollution]
	CIRCULAR	Recycling, reuse, and circularity	Explicit references to recycling, recyclability, reuse, material separation, recovery, or generic single-use and disposability meanings. Concrete products containing a single-use reference are coded according to their application context.	“Recycling” [recycling]; “Nicht recykelbar” [not recyclable]; “Gelber Sack” [yellow recycling bag]; “Wiederverwendbar” [reusable]; “Einweg” [single-use]
Use-related and economic meanings	UTILITY	Usefulness, practicality, and functional relevance	Evaluations of the material as practically useful, usable, comfortable, important, necessary, or indispensable. This category concerns the material’s practical benefit rather than the number or diversity of its possible applications.	“Praktisch” [practical]; “Nützlich” [useful]; “Wichtig” [important]; “Ohne geht es nicht” [cannot do without it]
	UBIQUITY	Everyday presence, prevalence, and frequent use	References to the material’s prevalence, frequency of use, widespread presence, everyday occurrence, or long-standing establishment. This category concerns how commonly the material	“Alltag” [everyday life]; “Überall” [everywhere]; “Viel verwendet” [widely used]; “Weit verbreitet” [widespread]

Higher-order category	Code	Category label	Definition and inclusion rule	Anchor examples (German [English])
			occurs rather than how many different purposes it can serve.	
	VERSATILE	Versatility and adaptability	References to the breadth, diversity, universality, or adaptability of possible uses. This category concerns the range of potential applications rather than the material's usefulness or actual prevalence.	"Vielseitig" [versatile]; "Vielfältig" [multifaceted]; "Universal einsetzbar" [universally applicable]; "Möglichkeiten" [possibilities]
	ECONOMIC	Cost and economic value	References to price, cost, fees, affordability, or economic value, including the cost of producing the material.	"Billig" [cheap]; "günstig" [inexpensive]; "billig in der Herstellung" [cheap to produce]
Material properties	DURABILITY	Durability, longevity, and ageing	References to temporal durability, service life, longevity, ageing, deterioration, or resistance over time or through environmental exposure. Generic strength, hardness, flexibility, and fracture behaviour without a temporal component are coded as MECHANICAL.	"Haltbar" [durable]; "langlebig" [long-lasting]; "unverwüstlich" [indestructible]; "schnell kaputt" [breaks quickly]
	MECHANICAL	Mechanical, structural, and form-related properties	Intrinsic structural or performance-related material properties describing behaviour under force, load, deformation, or handling, including hardness or softness, rigidity or flexibility, strength or fragility, weight, thickness, elasticity, and formability. Appearance, odour, surface texture, and thermal or other directly sensory qualities are excluded.	"Flexibel" [flexible]; "Formbar" [formable]; "Leicht" [lightweight]; "Hart" [hard]; "weich" [soft]; "zerbrechlich" [fragile]
	SENSORY	Visual and other sensory properties	Directly perceivable visual, olfactory, surface-related, thermal, or other sensory qualities of the material. Structural properties such as hardness	"Bunt" [colourful]; "durchsichtig" [transparent]; "Schlechter Geruch" [bad smell]; "Glatt" [smooth]; "Kalt" [cold]

Higher-order category	Code	Category label	Definition and inclusion rule	Anchor examples (German [English])
			or softness, elasticity, strength, weight, and formability are excluded.	
Material identity, composition, and origin	ARTIFICIAL	Artificiality and non-naturalness	References to perceived artificiality, synthetic character, non-naturalness, or inauthenticity.	“Künstlich” [artificial]; “Nicht natürlich” [not natural]; “Unecht” [not genuine]
	MATERIAL	Material types, physical forms, and composition	Concrete material classes, named material subtypes, constituents, trade abbreviations, physical material forms, or explicit references to composition. Generic paraphrases of the stimulus term such as “material” or “substance,” scientific or chemical concepts, and production processes are excluded.	“Verbundstoff” [composite material]; “Polyethylen” [polyethylene]; “Polyester” [polyester]; “PTFE” [PTFE]; “Duroplast” [thermoset]; “Elastomere” [elastomers]; “Granulat” [granulate]; “BPA” [BPA]; “PU/PA/TPU/PP/PY/Tef” [polymer abbreviations]
	FEEDSTOCK	Feedstocks, resources, and fossil origin	References to feedstocks, raw materials, natural resources, or the fossil origin of the material.	“Erdöl” [petroleum]; “Rohöl” [crude oil]; “Fossile Ressourcen” [fossil resources]; “Rohstoffe” [raw materials]
Science, production, and technological development	SCIENCE	Chemistry, science, and scientific terminology	References to scientific disciplines, chemical or molecular concepts, chemical structures, reactions, and other scientific terminology. Chemical reactions and conversion processes are included here; industrial shaping and manufacturing operations applied to materials are coded as PRODUCTION.	“Chemie” [chemistry]; “Moleküle” [molecules]; “Polymerisation” [polymerisation]; “Mehrfachverbindungen” [multiple bonds]; “Verbindung” [compound]; “chemische Verbindung” [chemical compound]; “Cracking” [cracking]
	PRODUCTION	Production, processing, and processability	References to industrial manufacturing, shaping, processing, processability, production machinery, or production at scale. Chemical reactions and conversion processes are coded as SCIENCE.	“Spritzguss” [injection moulding]; “Schmelzen” [melting]; “verarbeitbar” [processable]; “Massenproduktion” [mass production]

Higher-order category	Code	Category label	Definition and inclusion rule	Anchor examples (German [English])
	INNOVATION	Innovation, modernity, and future orientation	References to innovation, modernity, technological progress, development potential, invention, or future orientation.	“Innovation” [innovation]; “modern” [modern]; “Die fortschrittliche Technologie” [advanced technology]; “Zukunft” [future]
Applications, product forms, and use contexts	PACKAGING	Packaging and packaging materials	Explicit references to packaging, packaging materials, or clearly identifiable packaging products, including disposable, single-use, and returnable packaging. Generic container terms without a clearly identifiable packaging context are coded as EVERYDAY.	“Verpackung” [packaging]; “Joghurtbecher” [yoghurt cup]; “Pfandflaschen” [deposit bottles]; “Einwegflaschen” [single-use bottles]; “Tüten im Supermarkt” [bags in the supermarket]
	EVERYDAY	Everyday, household, and consumer uses	References to household, storage, clothing, food, leisure, furniture, toys, and other everyday-use or consumer applications. Generic container or product terms without an explicit packaging context are included here.	“Aufbewahrung” [storage]; “Tupperdosen” [food-storage containers]; “Kinderspielzeug” [children’s toys]; “Kleidung” [clothing]; “Flasche” [bottle]
	TECHNICAL	Technical, industrial, construction, and transport applications	References to specific technical, industrial, construction-related, digital, automotive, transport-related, or other specialised applications and devices. Generic production processes are coded as PRODUCTION.	“3-D-Drucker” [3D printer]; “Bauwesen” [construction]; “Computer” [computer]; “Industrielle Nutzung” [industrial use]
Human health, safety, and hygiene	HEALTHRISK	Human health risks and harmful substances	Explicit references to toxicity, harmful substances, or specific negative consequences for human health. Environmental harm without an explicit human-health target is coded as HARM.	“Ungesund” [unhealthy]; “Gesundheitsschädlich” [harmful to health]; “Krebserregend” [carcinogenic]; “Schadstoffe” [harmful substances]
	SAFETY	Safety, danger, general harmfulness, and trust	References to general danger, harmfulness, safety, trust, or regulatory safety when no	“Schädlich” [harmful]; “Gefährlich” [dangerous]; “Vertrauen” [trust]; “REACH” [REACH]

Higher-order category	Code	Category label	Definition and inclusion rule	Anchor examples (German [English])
			explicit environmental or human-health target is stated.	
	MED	Health, hygiene, medicine, and personal care	General or neutral references to health, cleanliness, hygiene, medicine, protection, cosmetics, or personal care, including corresponding products and application contexts. Explicit negative effects on human health are coded as HEALTHRISK.	“Gesundheit” [health]; “Medizin” [medicine]; “Sauber” [clean]; “Dreckig” [dirty]; “Kosmetik” [cosmetics]; “Einweghandschuhe” [disposable gloves]
Familiarity	UNFAMILIAR	Unfamiliarity, lack of knowledge, or no accessible association	Explicit unfamiliarity, lack of knowledge, uncertainty about the term, or an explicit statement that no association comes to mind.	“Keine Ahnung” [no idea]; “Kenne ich nicht” [I do not know it]; “Nie gehört” [never heard of it]; “keine” [none]; “nichts” [nothing]
	FAMILIAR	Familiarity and recognition	Explicit personal familiarity with or recognition of the term or material.	“Bekannt” [known]; “vertraut” [familiar]
Unspecified overall evaluation	POSITIVE	Unspecified positive evaluation	An unambiguous positive valence judgement without a specific substantive reason or target. When a specific reason or substantive meaning is stated, the corresponding content category takes precedence.	“Gut” [good]; “Cool” [cool]; “Schön” [beautiful]; “Gutes Material” [good material]
	NEGATIVE	Unspecified negative evaluation	An unambiguous negative valence judgement, rejection, or judgement of excess without a specific substantive reason or target. When a specific reason or substantive meaning is stated, the corresponding content category takes precedence.	“Schlecht” [bad]; “Problematisch” [problematic]; “Nicht gut” [not good]; “Viel zu viel” [far too much]; “Zu viel” [too much]

Higher-order category	Code	Category label	Definition and inclusion rule	Anchor examples (German [English])
Terminological and metalinguistic responses	EQUIVALENCE	Equivalence with other material terms	Explicit references to an equivalent, synonymous, or alternative material term. Definitions or explanations of the meaning of the stimulus word are coded as DEFINITION.	"Plastik" [plastic]; "Kunststoff" [synthetic material]; "Plaste" [plastic]; "Polymere" [polymers]
	DEFINITION	Definition, word meaning, and word decomposition	Explicit attempts to define or paraphrase the meaning of the stimulus term or to decompose the word into meaningful components. Generic paraphrases such as "material" or "substance" are included here when they function as explanations of the stimulus term.	"Poly" [poly]; "Mehrere Stoffe?" [several substances?]; "Poly meint verbunden, mehrere" [poly means connected/several]; "Material" [material]; "Stoff" [substance]
Other	OTHER	Other or non-classifiable association	Residual category for punctuation-only responses, idiosyncratic or unclear responses, and responses whose assignment to a substantive category would require inferring an unstated object, target, consequence, context, or intended spelling. It also includes interpretable content that falls outside the scope of the category system.	"." [period only]; "China" [China]; "Sommer" [summer];

Note. Anchor examples are verbatim German responses, followed by English translations in brackets. Coding was based exclusively on the manifest content of each response. Unstated objects, targets, consequences, contexts, or intended spellings were not inferred. When an association contained a specific substantive meaning and an evaluative component, the specific substantive category took precedence over an unspecified positive or negative evaluation.

F.2. Results

The number of associations provided per participant differed between the material-term conditions, Kruskal–Wallis $H(2) = 18.05$, $p < .001$, $\varepsilon^2 = .053$. Participants provided an average of $M = 3.25$ associations in the plastic condition ($SD = 1.70$), $M = 2.97$ in the synthetic-material condition ($SD = 1.82$) and $M = 2.31$ in the polymer condition ($SD = 1.47$). Holm-adjusted pairwise Mann–Whitney U-tests indicated that participants in the polymer condition provided fewer associations than participants in both the plastic condition, $U = 3278.00$, $p_{Holm} < .001$, and the synthetic-material condition, $U = 4009.00$, $p_{Holm} = .014$. The plastic and synthetic-material conditions did not differ, $U = 4621.50$, $p_{Holm} = .130$.

Nine coding categories differed significantly between the material-term conditions after Benjamini–Hochberg correction (Benjamini & Hochberg, 1995): general environmental and sustainability references, negative environmental meanings, recycling and circularity, feedstocks and fossil origin, science- and chemistry-related terminology, packaging, unfamiliarity, unspecified negative evaluations, and equivalence with other material terms. Full category frequencies and omnibus tests are reported in Table F.2. Holm-adjusted pairwise comparisons for the nine categories that remained significant after the omnibus Benjamini–Hochberg correction are shown in Table F.3. Negative environmental meanings followed a graded pattern, with plastic eliciting the category more frequently than synthetic material, and synthetic material more frequently than polymer. Polymer elicited fewer circularity, feedstock, and packaging associations than both other terms, but more science-related and unfamiliarity associations. Unspecified negative evaluations were more frequent for plastic than for either alternative term. Equivalence with another material term was substantially less frequent for plastic than for synthetic material or polymer.

The analyses were repeated using only each participant's first association. Six categories remained significant after Benjamini–Hochberg correction: general environmental and sustainability references, negative environmental meanings, science- and chemistry-related terminology, packaging, unfamiliarity, and equivalence with other material terms. Most importantly, negative environmental meanings remained most frequent for plastic, followed by synthetic material and polymer. Science-related terminology and unfamiliarity remained concentrated in the polymer condition. Complete results are reported in Table F.4. Holm-adjusted follow-up tests for the six significant first-association categories are reported in Table F.5.

<u>Reference:</u>

Benjamini, Y., and Hochberg, Y. (1995). Controlling the false discovery rate: a practical and powerful approach to multiple testing. *J. R. Stat. Soc. Ser. B 57*, 289–300. doi: 10.1111/j.2517-6161.1995.tb02031.x

Table F.2. Participant-Level Frequencies of Free-Association Categories by Material-Term Condition and Omnibus Tests.

Category	Plastic *n* (%)	Synthetic material *n* (%)	Polymer *n* (%)	$\chi^2(2)$	*p*	p_{BH}	Cramér's *V*
Environmental meanings:							
General environmental and sustainability references	16 (15.8)	5 (4.8)	4 (4.1)	11.57	.003	.011	.195
Negative environmental meanings	57 (56.4)	29 (27.9)	7 (7.1)	57.40	< .001	< .001	.435
Recycling, reuse, and circularity	23 (22.8)	15 (14.4)	3 (3.1)	16.62	< .001	< .001	.234
Use-related and economic meanings:							
Usefulness, practicality, and functional relevance	11 (10.9)	9 (8.7)	2 (2.0)	6.24	.044	.095	.144
Everyday presence, prevalence, and frequent use	7 (6.9)	6 (5.8)	1 (1.0)	—	.096	.167	.121
Versatility and adaptability	7 (6.9)	14 (13.5)	6 (6.1)	4.08	.130	.214	.116
Cost and economic value	7 (6.9)	2 (1.9)	1 (1.0)	—	.073	.137	.145
Material properties:							
Durability, longevity, and ageing	7 (6.9)	8 (7.7)	2 (2.0)	3.54	.170	.238	.108
Mechanical, structural, and form-related properties	10 (9.9)	18 (17.3)	9 (9.2)	3.86	.145	.226	.113
Visual and other sensory properties	7 (6.9)	12 (11.5)	6 (6.1)	2.30	.316	.402	.087
Material identity, composition, and origin:							
Artificiality and non-naturalness	4 (4.0)	7 (6.7)	10 (10.2)	3.02	.221	.295	.100
Material types, physical forms, and composition	3 (3.0)	13 (12.5)	13 (13.3)	7.66	.022	.056	.159
Feedstocks, resources, and fossil origin	11 (10.9)	10 (9.6)	1 (1.0)	8.50	.014	.044	.167
Science, production, and technological development:							
Chemistry, science, and scientific terminology	1 (1.0)	3 (2.9)	18 (18.4)	26.81	< .001	< .001	.297
Production, processing, and processability	4 (4.0)	3 (2.9)	0 (0.0)	—	.168	.238	.110
Innovation, modernity, and future orientation	3 (3.0)	3 (2.9)	3 (3.1)	—	1.000	1.000	.004
Applications, product forms, and use contexts:							
Packaging and packaging materials	22 (21.8)	17 (16.3)	2 (2.0)	17.64	< .001	< .001	.241
Everyday, household, and consumer uses	15 (14.9)	20 (19.2)	6 (6.1)	7.64	.022	.056	.159

Technical, industrial, construction, and transport applications	3 (3.0)	7 (6.7)	6 (6.1)	1.65	.437	.510	.074
Human health, safety, and hygiene:							
Human health risks and harmful substances	9 (8.9)	2 (1.9)	3 (3.1)	—	.048	.095	.146
Safety, danger, general harmfulness, and trust	6 (5.9)	3 (2.9)	2 (2.0)	—	.344	.418	.089
Health, hygiene, medicine, and personal care	2 (2.0)	2 (1.9)	3 (3.1)	—	.801	.863	.035
Familiarity and knowledge:							
Unfamiliarity, lack of knowledge, or no accessible association	1 (1.0)	2 (1.9)	20 (20.4)	33.99	< .001	< .001	.335
Familiarity and recognition	0 (0.0)	1 (1.0)	1 (1.0)	—	.770	.863	.058
Unspecified evaluations:							
Unspecified positive evaluation	2 (2.0)	3 (2.9)	3 (3.1)	—	.910	.944	.029
Unspecified negative evaluation	14 (13.9)	1 (1.0)	2 (2.0)	19.59	< .001	< .001	.254
Terminological and metalinguistic responses:							
Equivalence with other material terms	3 (3.0)	31 (29.8)	42 (42.9)	43.99	< .001	< .001	.381
Definition, word meaning, and word decomposition	2 (2.0)	6 (5.8)	11 (11.2)	7.30	.026	.061	.155
Other responses:							
Other or non-classifiable association	9 (8.9)	6 (5.8)	7 (7.1)	—	—	—	—

Note. Percentages indicate the proportion of participants who provided at least one association in the respective category. Pearson chi-square tests are reported unless expected-frequency assumptions were not met; in those cases, *p*-values are based on two-sided Fisher–Freeman–Halton exact tests. Unadjusted *p* values correspond to the reported omnibus tests. Benjamini–Hochberg-adjusted *p* values were computed across the 28 category tests. The OTHER category was reported descriptively and excluded from inferential testing.

Table F.3. Holm-Adjusted Pairwise Comparisons for Significant Categories.

Category	Plastic vs. synthetic material	Plastic vs. polymer	Synthetic material vs. polymer
General environmental and sustainability references	.025	.025	1.000
Negative environmental meanings	< .001	< .001	< .001
Recycling, reuse, and circularity	.151	< .001	.011
Feedstocks, resources, and fossil origin	.821	.015	.020
Chemistry, science, and scientific terminology	.621	< .001	< .001
Packaging and packaging materials	.375	< .001	< .001
Unfamiliarity, lack of knowledge, or no accessible association	1.000	< .001	< .001
Unspecified negative evaluation	< .001	.006	.612
Equivalence with other material terms	< .001	< .001	.058

Table F.4. Category Frequencies and Omnibus Tests Restricted to Participants' First Associations.

Category	Plastic *n* (%)	Synthetic material *n* (%)	Polymer *n* (%)	$\chi^2(2)$	*p*	p_{BH}	Cramér's *V*
Environmental meanings:							
General environmental and sustainability references	8 (7.9)	1 (1.0)	0 (0.0)	—	.002	.008	.207
Negative environmental meanings	28 (27.7)	13 (12.5)	1 (1.0)	29.95	< .001	< .001	.314
Recycling, reuse, and circularity	3 (3.0)	4 (3.8)	1 (1.0)	—	.544	.772	.073
Use-related and economic meanings:							
Usefulness, practicality, and functional relevance	3 (3.0)	1 (1.0)	0 (0.0)	—	.224	.457	.108
Everyday presence, prevalence, and frequent use	0 (0.0)	0 (0.0)	0 (0.0)	—	1.000	1.000	.000
Versatility and adaptability	2 (2.0)	6 (5.8)	0 (0.0)	—	.031	.109	.150
Cost and economic value	2 (2.0)	0 (0.0)	0 (0.0)	—	.214	.457	.115
Material properties:							
Durability, longevity, and ageing	3 (3.0)	4 (3.8)	0 (0.0)	—	.185	.457	.109
Mechanical, structural, and form-related properties	3 (3.0)	5 (4.8)	6 (6.1)	—	.585	.780	.061
Visual and other sensory properties	2 (2.0)	2 (1.9)	1 (1.0)	—	1.000	1.000	.034
Material identity, composition, and origin:							
Artificiality and non-naturalness	4 (4.0)	4 (3.8)	4 (4.1)	—	1.000	1.000	.005
Material types, physical forms, and composition	2 (2.0)	6 (5.8)	6 (6.1)	—	.313	.503	.089
Feedstocks, resources, and fossil origin	5 (5.0)	5 (4.8)	1 (1.0)	—	.246	.458	.097
Science, production, and technological development:							
Chemistry, science, and scientific terminology	1 (1.0)	2 (1.9)	11 (11.2)	—	< .001	.006	.218
Production, processing, and processability	0 (0.0)	1 (1.0)	0 (0.0)	—	1.000	1.000	.080
Innovation, modernity, and future orientation	0 (0.0)	0 (0.0)	1 (1.0)	—	.323	.503	.083
Applications, product forms, and use contexts:							
Packaging and packaging materials	7 (6.9)	2 (1.9)	0 (0.0)	—	.009	.040	.171
Everyday, household, and consumer uses	9 (8.9)	7 (6.7)	3 (3.1)	2.95	.228	.457	.099

Technical, industrial, construction, and transport applications	2 (2.0)	2 (1.9)	0 (0.0)	—	.552	.772	.080
Human health, safety, and hygiene:							
Human health risks and harmful substances	9 (8.9)	2 (1.9)	3 (3.1)	—	.048	.095	.146
Safety, danger, general harmfulness, and trust	6 (5.9)	3 (2.9)	2 (2.0)	—	.344	.418	.089
Health, hygiene, medicine, and personal care	2 (2.0)	2 (1.9)	3 (3.1)	—	.801	.863	.035
Familiarity and knowledge:							
Unfamiliarity, lack of knowledge, or no accessible association	1 (1.0)	2 (1.9)	17 (17.3)	27.21	< .001	< .001	.300
Familiarity and recognition	0 (0.0)	0 (0.0)	0 (0.0)	—	1.000	1.000	.000
Unspecified evaluations:							
Unspecified positive evaluation	2 (2.0)	3 (2.9)	2 (2.0)	—	1.000	1.000	.028
Unspecified negative evaluation	3 (3.0)	0 (0.0)	0 (0.0)	—	.069	.201	.141
Terminological and metalinguistic responses:							
Equivalence with other material terms	2 (2.0)	25 (24.0)	34 (34.7)	34.61	< .001	< .001	.338
Definition, word meaning, and word decomposition	1 (1.0)	5 (4.8)	7 (7.1)	—	.072	.201	.124
Other responses:							
Other or non-classifiable association	2 (2.0)	3 (2.9)	2 (2.0)	—	—	—	—

Note. Percentages indicate the proportion of participants who provided at least one association in the respective category. Pearson chi-square tests are reported unless expected-frequency assumptions were not met; in those cases, *p*-values are based on two-sided Fisher–Freeman–Halton exact tests. Unadjusted *p* values correspond to the reported omnibus tests. Benjamini–Hochberg-adjusted *p* values were computed across the 28 category tests. The OTHER category was reported descriptively and excluded from inferential testing.

Table F.5. Holm-Adjusted Pairwise Comparisons for Significant Categories in the First-Association Sensitivity Analysis.

Category	Plastic vs. synthetic material	Plastic vs. polymer	Synthetic material vs. polymer
General environmental and sustainability references	.035	.020	1.000
Negative environmental meanings	.008	< .001	.003
Chemistry, science, and scientific terminology	1.000	.007	.017
Packaging and packaging materials	.195	.042	.498
Unfamiliarity, lack of knowledge, or no accessible association	1.000	< .001	< .001
Equivalence with other material terms	< .001	< .001	.122

G. Study 2: Manipulation Materials and Checks

Participants received the following instruction:

Auf der nächsten Seite erhalten Sie einen kurzen Informationstext zu einem Material, das in verschiedenen technischen Anwendungen zum Einsatz kommt. Bitte lesen Sie den Text aufmerksam durch. Anschließend bitten wir Sie, uns Ihre Einschätzung zum Material und zu seiner Verwendung mitzuteilen.

***WICHTIG**: Bitte lesen Sie den Text wirklich aufmerksam durch! Ihre Einschätzung der Inhalte ist im weiteren Verlauf sehr wichtig.*

[On the next page, you will receive a short information text about a material used in various technical applications. Please read the text carefully. Afterwards, we will ask you to provide your assessment of the material and its use.

***IMPORTANT**: Please read the text very carefully. Your assessment of the content will be very important later in the survey.]*

The instruction was followed by the manipulation texts presented in Table G.1. In the survey, [TERM] was replaced by the assigned material term: *Plastik [plastic]*, *Kunststoff [synthetic material]*, or *Polymer [polymer]*. The experimental information condition provided application-specific explanatory information about the material's functional role in green technologies and its contribution to long-term performance and resource use. The control information condition described forms and areas of application without providing the same functional explanation. After reading the text, participants completed an attention check, a forced-choice text-comprehension check, and an open material manipulation check (see Table G.2).

Table G.1. Manipulation Texts

	German original	English translation
Introductory sentence presented in both conditions	In vielen grünen Technologien wie Solarpanels oder Windkraftanlagen wird als Material [TERM] verwendet. In der Öffentlichkeit wird dieses Material unterschiedlich bewertet, und generell kommt es in sehr vielen Bereichen zum Einsatz.	In many green technologies, such as solar panels or wind turbines, [TERM] is used as a material. The public evaluates this material in different ways, and it is generally used in many areas.
Green-technology-specific benefit information condition	[TERM] wird vor allem deshalb in grünen Technologien eingesetzt, weil dieses Material bestimmte Eigenschaften aufweist – etwa Langlebigkeit, Stabilität, Witterungsbeständigkeit, geringes Gewicht und technologische Vielseitigkeit. Diese Eigenschaften sind entscheidend dafür, dass z.B. Solarpanels auch bei Sturm, Hitze oder Frost zuverlässig arbeiten können und Windkraftanlagen über viele Jahre hinweg stabil Strom erzeugen.<br>[TERM] einzusetzen trägt also dazu bei, dass grüne Technologien langfristig sicher und effizient funktionieren – und damit einen wichtigen Beitrag zu einer nachhaltigen, verantwortungsbewussten Nutzung von Ressourcen leisten.	[TERM] is used in green technologies primarily because this material has certain properties, such as durability, stability, weather resistance, low weight, and technological versatility. These properties are crucial for ensuring that, for example, solar panels can operate reliably even during storms, heat, or frost, and that wind turbines can generate electricity steadily over many years.<br>Using [TERM] therefore contributes to green technologies functioning safely and efficiently over the long term, and thereby makes an important contribution to the sustainable and responsible use of resources.
General application information condition	[TERM] wird in grünen Technologien in unterschiedlicher Form verarbeitet – etwa als feste, flache oder flexible Komponente. Auch in zahlreichen anderen technischen und industriellen Produkten findet dieses Material Verwendung, z. B. in der Produktion von Gehäusen, Bauteilen oder Oberflächenmaterialien. Dabei kann es sich z.B. auch um Einzelteile in elektronischen Geräten oder Verkleidungen in Maschinen handeln.<br>[TERM] wird bereits seit mehreren Jahrzehnten eingesetzt und hat sich über die Zeit in seinen Erscheinungsformen und Anwendungsbereichen stetig verändert – von einfachen Haushaltsprodukten bis hin zu hochspezialisierten technischen Anwendungen.	[TERM] is processed in green technologies in different forms, for example as a solid, flat, or flexible component. This material is also used in numerous other technical and industrial products, for example in the production of housings, components, or surface materials. It may also be used as individual parts in electronic devices or as coverings in machines.<br>[TERM] has been used for several decades and has steadily changed over time in its forms and areas of application, ranging from simple household products to highly specialised technical applications.

Table G.2. Attention, Comprehension, and Manipulation Checks

German original	English translation
Item 1: Wie aufmerksam haben Sie den Text gelesen?<br>1 = *nicht aufmerksam* bis 7 = *sehr aufmerksam*	Item 1: How attentively did you read the text?<br>1 = *not attentively* to 7 = *very attentively*
Item 2: Bevor wir Sie um Ihre Einschätzung zum im Text beschriebenen Material und Technologien bitten, interessiert uns, was bei Ihnen hängen geblieben ist. Worum ging es im Informationstext?<br>a) Der Text stellte die Verbreitung verschiedener Energiequellen in Deutschland und Österreich vor.<br>b) Der Text stellte die Vor- und Nachteile von Windkraft gegenüber Photovoltaik dar.<br>c) Der Text erklärte, wie man Windkraftanlagen selbst bauen kann.<br>d) Der Text beschrieb die Rolle eines bestimmten Materials in Technologien wie Solarpanels oder Windkraftanlagen.<br>e) Der Text erklärte, wie sich technische Materialien auf Stromkosten auswirken.	Item 2: Before we ask you to provide your assessment of the material and technologies described in the text, we are interested in what you remember. What was the information text about?<br>a) The text presented the prevalence of different energy sources in Germany and Austria.<br>b) The text presented the advantages and disadvantages of wind power compared with photovoltaics.<br>c) The text explained how to build wind turbines oneself.<br>d) The text described the role of a specific material in technologies such as solar panels or wind turbines.<br>e) The text explained how technical materials affect electricity costs.
Item 3: Welches Material wurde im Informationstext genannt? Geben Sie einfach ein, woran Sie sich erinnern – die Schreibweise ist nicht wichtig.	Item 3: Which material was mentioned in the information text? Please simply enter what you remember; spelling is not important.

Note. Item 1 was rated on a 7-point scale. Item 2 was a single-choice comprehension check; response option d) was the correct answer. Item 3 was an open-ended manipulation check.

H. Study 2: Measurement Model

The theoretical five-factor model distinguished perceived material usefulness, perceived material sustainability, perceived technology usefulness, perceived technology sustainability, and intention to use. This model showed acceptable fit (see Table H.1). However, several latent factors were substantially correlated. The highest latent correlation was observed between perceived technology usefulness and perceived technology sustainability, $r = .852$. Perceived technology usefulness was also strongly correlated with intention to use, $r = .757$. The correlation between perceived material usefulness and perceived material sustainability was $r = .612$. These associations were theoretically plausible but warranted additional tests of factorial distinctiveness. We therefore compared the five-factor model with more restrictive models in which conceptually related and empirically correlated constructs were combined.

As shown in Table H.1, all restricted models fitted the data substantially worse than the theoretical five-factor model. The comparisons therefore supported retaining the five constructs as separate but related dimensions in the subsequent path analyses, while acknowledging their strong associations.

Table H.1. Confirmatory Factor Analyses for the Measured Constructs in Study 2.

Model	$\chi^2(df)$	CFI	TLI	RMSEA	SRMR	AIC	$\Delta\chi^2(df)$
Five-factor model	445.50 (142)	.960	.952	.076	.041	541.50	—
Material usefulness + sustainability collapsed	1201.96 (146)	.860	.837	.141	.083	1289.96	756.46 (4)
Technology usefulness + sustainability collapsed	826.72 (146)	.910	.895	.113	.048	914.72	381.22 (4)
Technology usefulness + intention to use collapsed	1016.20 (146)	.885	.865	.128	.055	1104.20	570.70 (4)

Note. The CFA was based on $N = 367$ complete cases. $\Delta\chi^2$ values refer to comparisons with the five-factor model. All $\Delta\chi^2$ tests were significant at $p < .001$.

I. Study 2: Direct Experimental Effects

Table I.1 reports Sidak-corrected pairwise comparisons for the proximal material-perception outcomes. Because the material term × information condition interaction for intention to use was significant but small, we examined the simple effects of information condition within each material-term condition. None of the simple effects reached significance (Table I.2). The descriptive pattern suggested a more positive effect of green-technology-specific benefit information in the plastic and synthetic material conditions, but not in the polymer condition. Given the small interaction and the absence of significant simple effects, this interaction was interpreted cautiously.

Prior green-technology use was positively associated with more favourable technology perceptions, $r = .14$, $p = .005$ for PTS and $r = .17$, $p < .001$ for PTU, and with stronger ITU, $r = .22$, $p < .001$. Therefore, as a robustness check, we repeated the direct experimental analyses while controlling for prior green-technology use (Table I.3). The conclusions remained unchanged. Material terminology remained significantly associated with PMS and PMU, and information condition also remained a small but significant predictor of both material perceptions. The material terminology × information condition interactions remained non-significant for both material perceptions. At the more distal level, neither material terminology nor information condition significantly predicted technology perceptions after prior use was controlled for. For ITU, the main effects remained non-significant, whereas the small interaction remained significant. Prior green-technology use was positively associated with all outcomes.

Table I.1. Sidak-Corrected Pairwise Comparisons for Material-Term Effects on Proximal Material Perceptions

Outcome	Comparison	M_{diff}	*SE*	*p*	Sidak-adjusted 95% CI
Perceived material sustainability	Synthetic material – plastic	0.39	0.17	.068	[−0.02, 0.81]
	Polymer – plastic	1.50	0.18	< .001	[1.07, 1.94]
	Polymer – synthetic material	1.11	0.18	< .001	[0.69, 1.53]
Perceived material usefulness	Synthetic material – plastic	0.35	0.15	.070	[−0.02, 0.72]
	Polymer – plastic	0.76	0.16	< .001	[0.38, 1.15]
	Polymer – synthetic material	0.41	0.16	.026	[0.04, 0.79]

Note. Positive values indicate higher scores for the first-named condition.

Table I.2. Simple Effects of Information Condition on Intention to Use by Material Term

Material term	M_{diff}	*SE*	95% CI	*F*	*df*	*p*	partial η^2
Plastic	0.69	0.39	[−0.08, 1.46]	3.12	1, 384	.078	.008
Synthetic material	0.61	0.36	[−0.11, 1.33]	2.81	1, 384	.095	.007
Polymer	−0.54	0.40	[−1.33, 0.24]	1.85	1, 384	.174	.005

Note. Positive mean differences indicate higher intention to use in the green-technology-specific benefit information condition than in the general application information condition.

Table I.3. Factorial ANCOVAs Controlling for Prior Green-Technology Use

Outcome	Effect	*F*	*df*	*p*	partial η^2
Perceived material sustainability	Prior green-technology use	4.15	1, 383	.042	.011
	Material term	38.07	2, 383	< .001	.166
	Information condition	7.04	1, 383	.008	.018
	Material term × information condition	0.84	2, 383	.431	.004
Perceived material usefulness	Prior use	8.83	1, 383	.003	.023
	Material term	11.69	2, 383	< .001	.058
	Information condition	5.03	1, 383	.026	.013
	Material term × information condition	0.24	2, 383	.787	.001
Perceived technology sustainability	Prior green-technology use	6.95	1, 383	.009	.018
	Material term	1.30	2, 383	.273	.007
	Information condition	3.01	1, 383	.084	.008
	Material term × information condition	0.87	2, 383	.421	.005
Perceived technology usefulness	Prior green-technology use	9.55	1, 383	.002	.024
	Material term	1.10	2, 383	.333	.006
	Information condition	2.93	1, 383	.088	.008
	Material term × information condition	1.42	2, 383	.242	.007
Intention to use	Prior green-technology use	19.45	1, 383	< .001	.048
	Material term	0.47	2, 383	.626	.002
	Information condition	0.46	1, 383	.496	.001
	Material term × information condition	3.45	2, 383	.033	.018

Note. Material and technology perception variables were measured on 7-point scales; intention to use was measured on a 10-point scale.

J. Study 2: Path Model

Material-to-technology transfer models were estimated using maximum likelihood estimation. Material terminology was represented by two dummy variables: synthetic material versus plastic, and polymer versus plastic, with plastic as the reference category. To obtain the additional polymer-versus-synthetic-material contrast for the indirect effects, the model was re-estimated with synthetic material as the reference category. Information condition was coded so that green-technology-specific benefit information was compared with the general application information condition.

Interaction terms between material terminology and information condition were not included in the transfer models. The path models were designed to examine indirect main-effect patterns linking the experimental conditions to ITU through material and technology perceptions. This specification was consistent with the factorial analyses, which showed main effects of material term and information condition on PMS and PMU, but no interaction on these proximal outcomes. The small interaction observed for ITU was distal, was not accompanied by significant simple effects, and was therefore interpreted cautiously rather than used to define the path-model structure.

The hypothesised model specified two serial transfer pathways. The sustainability pathway linked material terminology and information condition to ITU through PMS and PTS. The usefulness pathway linked the experimental conditions to ITU through PMU and PTU. Direct paths from PMS and PMU to ITU were retained to account for residual associations beyond technology perceptions. The model was estimated as a full partial-mediation model that also included direct paths from the experimental predictors to PTS, PTU, and ITU.

The material-term dummy variables and the information-condition variable were allowed to covary. Residual covariances were specified between PMS and PMU, and between PTS and PTU, to account for shared unexplained variance within the material-perception and technology-perception domains.

The hypothesised model showed inadequate global fit, $\chi^2(2) = 38.98$, $p < .001$, CFI = .969, TLI = .568, RMSEA = .218, SRMR = .058. Model diagnostics indicated an omitted association from PMU to PTS. We therefore estimated a data-informed extended full partial-mediation model that retained the original paths and covariances and added a path from PMU to PTS. This cross-domain path was theoretically plausible because in the green technology context, materials perceived as useful, durable, and functionally enabling may also make the corresponding technologies appear more viable, efficient, and sustainable. The extended full partial-mediation model showed good fit, $\chi^2(1) = 3.06$, $p = .081$, CFI = .998, TLI = .952, RMSEA = .073, SRMR = .014. Accordingly, adding the cross-domain path from PMU to PTS significantly improved model fit, $\Delta\chi^2(1) = 35.92$, $p < .001$.

In the extended full partial-mediation model, the direct paths from the experimental predictors to PTS, PTU, and ITU were not statistically significant after material and technology perceptions were included. We therefore compared this model with a reduced extended model in which these nine direct paths were constrained to zero. The reduced model showed similarly good fit, $\chi^2(10) = 13.14$, $p = .216$, CFI = .997, TLI = .993, RMSEA = .028, SRMR = .033, $\Delta\chi^2(9) = 10.09$, $p = .343$, and was retained as the final, data-informed model.

In this final model, material terminology and information condition were indirectly associated with ITU through material and technology perceptions; direct paths from PMS and PMU to ITU remained included. Full path coefficients for the final model are reported in Table J.1.

We also repeated the final model with prior green-technology use included as an additional covariate predicting all endogenous variables. Model fit remained good, $\chi^2(10) = 13.38$, $p = .203$, CFI = .997, TLI = .990, RMSEA = .029, and the core transfer paths remained substantively unchanged. Material terms and information condition predicted material perceptions, PMU predicted both PTU and PTS, and PTU remained the strongest predictor of ITU. Prior green-technology use showed positive associations with PMS, $B = 0.31$, $SE = 0.15$, $\beta = .094$, $p = .041$; PMU, $B = 0.40$, $SE = 0.13$, $\beta = .145$, $p = .003$; PTU, $B = 0.29$, $SE = 0.13$, $\beta = .099$, $p = .029$; and ITU, $B = 0.47$, $SE = 0.16$, $\beta = .101$, $p = .003$. The association with PTS did not reach significance, $B = 0.26$, $SE = 0.15$, $\beta = .082$, $p = .080$.

The indirect effects in the covariate model were also consistent with the main analysis (Table J.2). The sustainability, usefulness, and cross-domain pathways were significant for synthetic material versus plastic, polymer versus plastic, and information condition. Total serial indirect effects were significant for all three experimental predictors, with the largest effect again observed for polymer versus plastic.

Table J.1. Path Coefficients (Study 2).

Outcome	Predictor	*B*	*SE*	95% CI	β	*p*
PMS	Synthetic material vs. plastic	0.39	0.17	[0.05, 0.75]	.122	.022
	Polymer vs. plastic	1.49	0.18	[1.13, 1.83]	.445	< .001
	Information condition	0.43	0.14	[0.15, 0.71]	.137	.003
PMU	Synthetic material vs. plastic	0.35	0.15	[0.04, 0.67]	.130	.022
	Polymer vs. plastic	0.75	0.16	[0.44, 1.05]	.269	< .001
	Information condition	0.33	0.13	[0.08, 0.58]	.128	.009
PTS	PMS	0.12	0.04	[0.05, 0.19]	.129	< .001
	PMU	0.35	0.06	[0.21, 0.50]	.311	< .001
PTU	PMU	0.48	0.05	[0.34, 0.61]	.458	< .001
ITU	PTS	0.31	0.09	[0.07, 0.58]	.209	< .001
	PTU	0.85	0.10	[0.55, 1.12]	.536	< .001
	PMU	0.19	0.08	[0.03, 0.36]	.113	.016
	PMS	−0.17	0.06	[−0.28, −0.07]	−.124	.005

Note. Confidence intervals are 95% bias-corrected bootstrap confidence intervals based on 5,000 bootstrap samples. Plastic was the reference category for material terminology. PMS = Perceived material sustainability; PMU = Perceived material usefulness; PTS = Perceived technology sustainability; PTU = perceived technology usefulness; ITU = Intention to use green technology.

Table J.2. Bootstrap Indirect Effects Controlling for Prior Green-Technology Use

Pathway	Synthetic material vs. plastic	Polymer vs. plastic	Polymer vs. synthetic material	Green-technology-specific benefit information
Sustainability pathway	0.014<br>[0.002, 0.046]	0.056<br>[0.014, 0.136]	0.042<br>[0.010, 0.104]	0.015<br>[0.003, 0.047]
Usefulness pathway	0.127<br>[0.013, 0.307]	0.295<br>[0.162, 0.527]	0.168<br>[0.070, 0.333]	0.114<br>[0.024, 0.263]
Cross-domain pathway	0.034<br>[0.005, 0.114]	0.079<br>[0.021, 0.204]	0.045<br>[0.010, 0.133]	0.030<br>[0.004, 0.098]
Total serial indirect effect	0.175<br>[0.021, 0.369]	0.430<br>[0.270, 0.630]	0.255<br>[0.112, 0.424]	0.159<br>[0.034, 0.319]

Note. Values are unstandardised indirect effects with 95% bias-corrected bootstrap confidence intervals in brackets. Confidence intervals were based on 5,000 bootstrap samples. The sustainability pathway refers to the indirect effect through perceived material sustainability → perceived technology sustainability → intention to use. The usefulness pathway refers to the indirect effect through perceived material usefulness → perceived technology usefulness → intention to use. The cross-domain pathway refers to the indirect effect through perceived material usefulness → perceived technology sustainability → intention to use. The total serial indirect effect represents the sum of these serial indirect pathways.
